# Synthetic Transition from Thiourea-Based Compounds to Tetrazole Derivatives: Structure and Biological Evaluation of Synthesized New *N*-(Furan-2-ylmethyl)-1*H*-tetrazol-5-amine Derivatives

**DOI:** 10.3390/molecules26020323

**Published:** 2021-01-10

**Authors:** Daniel Szulczyk, Anna Bielenica, Piotr Roszkowski, Michał A. Dobrowolski, Wioletta Olejarz, Sebastian Kmiecik, Małgorzata Podsiad, Marta Struga

**Affiliations:** 1 Chair and Department of Biochemistry, Medical University of Warsaw, 02-097 Warszawa, Poland; anna.bielenica@wum.edu.pl (A.B.); malgorzata.podsiad@wum.edu.pl (M.P.); mstruga@wum.edu.pl (M.S.); 2 Faculty of Chemistry, University of Warsaw, Pasteura 1, 02-093 Warszawa, Poland; roszkowski@chem.uw.edu.pl (P.R.); miked@chem.uw.edu.pl (M.A.D.); 3 Department of Biochemistry and Pharmacogenomics, Faculty of Pharmacy, Medical University of Warsaw, 02-097 Warszawa, Poland; wioletta.olejarz@wum.edu.pl; 4 Biological and Chemical Research Centre, Faculty of Chemistry, University of Warsaw, 02-089 Warsaw, Poland; sekmi@chem.uw.edu.pl

**Keywords:** antimicrobial, antibacterial, tetrazole, cytotoxicity, crystal structure

## Abstract

Twelve novel derivatives of *N*-(furan-2-ylmethyl)-1*H*-tetrazol-5-amine were synthesized. For obtained compound **8**, its corresponding substrate single crystals were isolated and X-ray diffraction experiments were completed. In the initial stage of research, in silico structure-based pharmacological prediction was conducted. All compounds were screened for their antibacterial and antimycobacterial activities using standard and clinical strains. The cytotoxic activity was evaluated against a panel of human cancer cell lines, in contrast to normal (HaCaT) cell lines, by using the MTT method. All examined derivatives were found to be noncytotoxic against normal cell lines. Within the studied group, compound **6** showed the most promising results in antimicrobial studies. It inhibited four hospital *S. epidermidis* rods’ growth, when applied at the amount of 4 µg/mL. However, the most susceptible to the presence of compound **6** was *S. epidermidis T 5501 851/19* clinical strain, for which the MIC value was only 2 µg/mL. Finally, a pharmacophore model was established based on lead compounds from this and our previous work.

## 1. Introduction

Recently, there is a need for research and development of new antimicrobials. Past years showed an increasing number of patients suffering from antibiotic-resistant bacterial strains. Antimicrobial resistance is the ability of microorganisms such as bacteria, fungi, or protozoans to grow despite exposure to antimicrobial substances designed to inhibit their growth.

A number of studies were engaged to help solve this issue. Scientists worldwide are trying to identify new natural antimicrobials isolated from plants or design new small molecules that might serve as future antimicrobial medicines. In some of the published papers it can be found that thiourea derivatives are recognized as promising antimicrobial agents [1,2,3,4,5,6,7,8]. Thiourea derivatives are relatively easy to synthesize and can be used for further structural modifications. One of the interesting scaffolds that can be obtained from them is the tetrazole ring. The 1,5-disubstituted tetrazoles can be generated by oxidative desulfurization of 1,3-disubstituted thioureas. This method acquires external nucleophile—sodium azide and leads to corresponding 1,5-disubstituted tetrazoles. Mercury salts serve as desulfurization agents.

**Figure 1:**
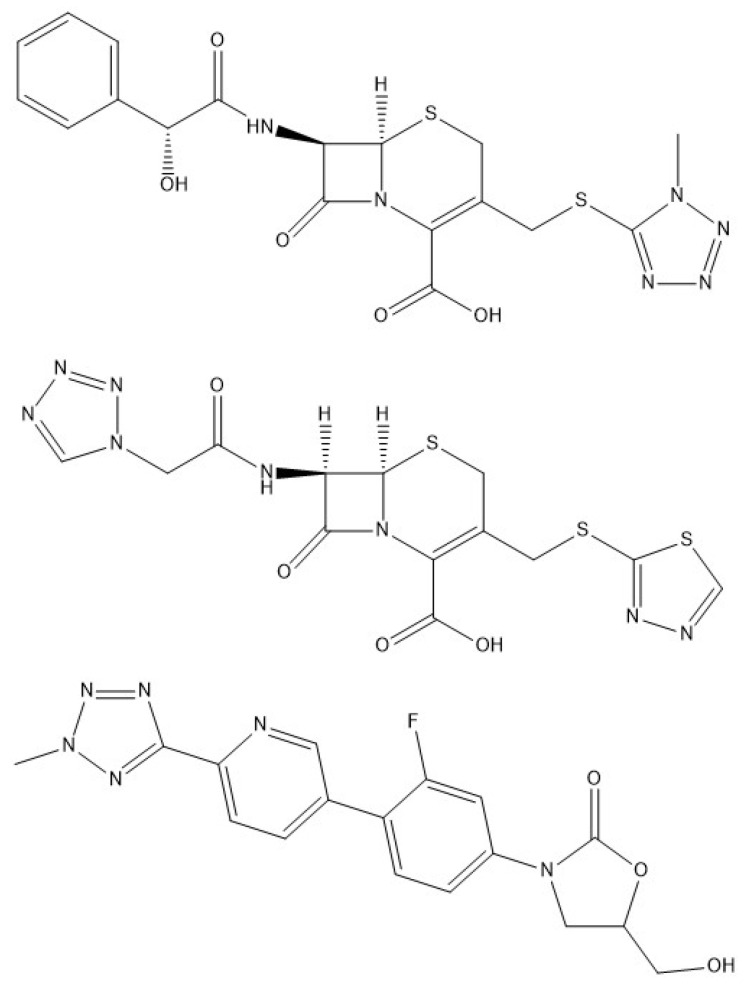
Structures of tetrazole-based antibiotics—Cefamandole, Ceftezole, and Tadalizolid, respectively.

In past years a few interesting studies of tetrazole-based compounds possessing antimicrobial activity were published [9,10,11]. Moreover, clinicians have already used tetrazole-derived antimicrobial drugs such as Cefamandole, Ceftezole, both second-generation broad-spectrum cephalosporin antibiotics and an oxazolidinone-class antibiotic Tadalizolid (Figure 1).

We can also find very promising studies concerning tetrazoles as antibacterial agents. Dişli et al. reported the successful synthesis and antimicrobial activity of new 6-dimethoxypyrimidine compounds incorporating a 1*H*-tetrazol-5-ylthio moiety. The synthesized compounds were evaluated for in vitro antibacterial activity against Gram-positive (*Staphylococcus aureus* ATCC 25923, *Streptococcus agalactiae* from Institute of Pasteur, 55118) and Gram-negative (*Klebsiella pneumoniae* ATCC 700603) bacteria strains. The results showed that some of these compounds exhibited as good antibacterial activity as that of standard antibiotics Penicillin, Ampicillin, and Erythromycin [9]. In another paper, tetrazole-1,2,3-triazole hybrids displayed promising in vitro antibacterial and antifungal activities against a panel of clinically important bacteria such as *S. aureus, Bacillus subtilis* or *Escherichia coli*, and six of them were comparable to Gatifloxacin [10]. Other research showed that a series of tetrazole-norfloxacin conjugates and their derivatives were found active against various strains of *S. aureus* including drug-resistant strains MRSA and Gentamycin-resistant *S. aureus* (GRSA), *E. coli* and *K. pneumoniae*. The majority of them showed great potency against tested Gram-positive and Gram-negative pathogens, with MIC values ranging from 0.39 to 50 mg/mL, and they were comparable or more effective than Ciprofloxacin and Gentamycin against staphylococci [11]. Above-mentioned examples and many more are well disclosed in a review of tetrazole-based compounds with antimicrobial activity [12].

During previous years, our team also participated in research for new antimicrobial agents built on a tetrazole scaffold [13,14,15]. On the other hand, using our experience in working with other heterocyclic skeleton-based compounds [16,17,18,19] we have decided to design and synthesize a series of heterocyclic scaffold tetrazole hybrids [20]. The best results were found for *N*-(furan-2-ylmethyl)-1*H*-tetrazol-5-amine derivatives, therefore it was obvious to prepare new compounds possessing that moiety and evaluate them as antimicrobial agents.

## 2. Results and Discussion

### 2.1. In Silico Structure-based Pharmacological Prediction

#### 2.1.1. Antibacterial Activity

Using AntiBac-Pred [21] web services of the Way2Drug platform, activity against Gram-positive and Gram-negative bacteria was predicted for twelve synthesized compounds. It was found that the whole group might be active against *Mycobacterium tuberculosis* (confidence values were ranging from 0.1861 to 0.0123) and *Sarcina* (confidence values were ranging from 0.2401 to 0.2197). It should be mentioned that interesting probability scores were found for compounds **7** (confidence value 0.4039), **10** (confidence value 0.2411), and **12** (confidence value 0.1718) for activity against *Staphylococcus aureus subsp. aureus* MW2 resistant strain.

#### 2.1.2. Toxicological Parameters

The pkCSM [22] software was used for the evaluation of toxicological parameters in silico. Evaluation revealed that none of synthesized compounds were positive in the AMES test, therefore recognizing it as non-carcinogenic. Furthermore, all derivatives were found not to be hERG I/II (the human Ether-à-go-go-Related Gene) inhibitors, which are the principal causes of acquire long QT syndrome. Flathead minnow larvae test revealed the low environmental hazard in the case of all derivatives, since all results were higher than 0.5 nM over two times. Results are consistent in terms of the skin sensitization test, as all compounds should not develop this effect. The maximum recommended tolerated dose (MRTD) should be considered as low. In most of the test results (Table 1), it could be stated that they are consistent along synthesized compounds.

### 2.2. Chemistry

**Scheme 1:**
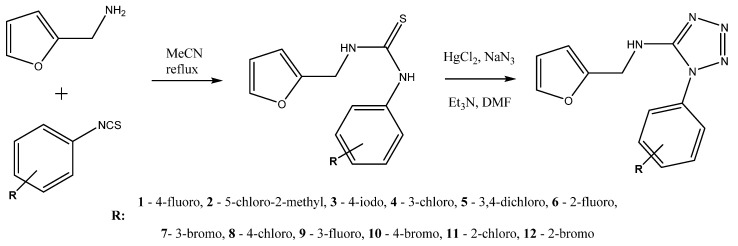
Transition from thiourea-based substrates to tetrazole derivatives.

Thiourea substrates were obtained in our past project. Synthesis, physicochemical and biological evaluation were extensively described in the published paper [23]. The main goal in the current design was to replace the thiourea arrangement with a tetrazole scaffold. Twelve new *N*-(furan-2-ylmethyl)-1*H*-tetrazol-5-amine derivatives were synthesized according to the well-known procedure [15]. Reaction conditions are depicted below (Scheme 1). All compounds were obtained in good yields, ranging from 79% to 92%. Yield differences are linked to the amount of unreacted substrates in each reaction.

**Figure 2:**
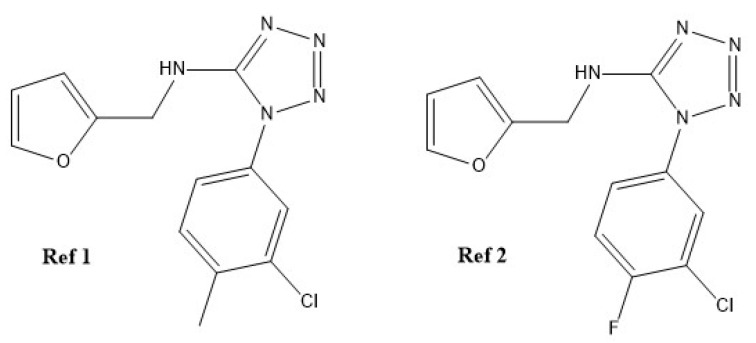
The structure of 1-(3-chloro-4-methylphenyl)-*N*-(furan-2-ylmethyl)-1*H*-tetrazol-5-amine (**Ref 1**) and 1-(3-chloro-4-fluorophenyl)-*N*-(furan-2-ylmethyl)-1*H*-tetrazol-5-amine (**Ref 2**).

It should be stated that two additional compounds possessing a furan-2yl-methyl moiety were synthesized on an earlier stage of the research and investigated for antimicrobial activity (Figure 2) [20].

Both (**Ref 1** and **Ref 2**) were synthesized in the same manner as the new twelve derivatives. These compounds were recognized as leading structures, possessing the highest antimicrobial activity, thus encouraging our team to design different furan-2yl-methyl tetrazole hybrids for further evaluation. In subsequent parts of the paper, these compounds will be used as the reference for newly synthesized derivatives.

### 2.3. X-ray Studies

The structure of 1-(4-chlorophenyl)-*N*-(furan-2-ylmethyl)-1*H*-tetrazol-5-amine **8** and 1-(4-chlorophenyl)-3-(furan-2-ylmethyl)thiourea **8a** were determined by X-ray diffraction on a single crystal. The thiourea-based substrate was characterized in previously published papers [23,24], but no X-ray studies were presented. Both (**8a**) and (**8**) crystallize in a monoclinic system, with one molecule in an asymmetric part of the unit cell—the former in centrosymmetric space group P2_1_/c, the latter in non-centrosymmetric P2_1_. Crystal data and structure refinement were recorded (Table S1, X-ray studies file, Supplementary Materials).

**Figure 3:**
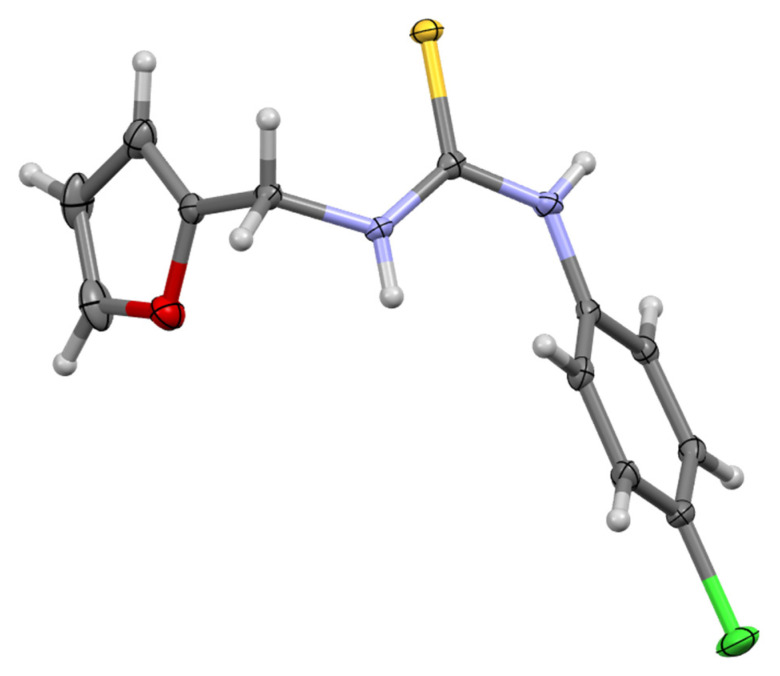

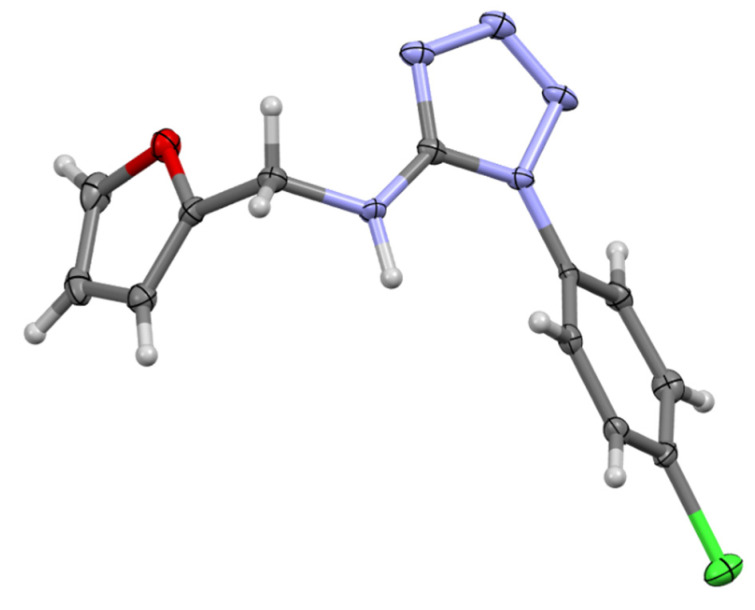
The molecular structure of (**8a, top**) and (**8, bottom**) showing displacement ellipsoids at the 50% probability level.

**Figure 4:**
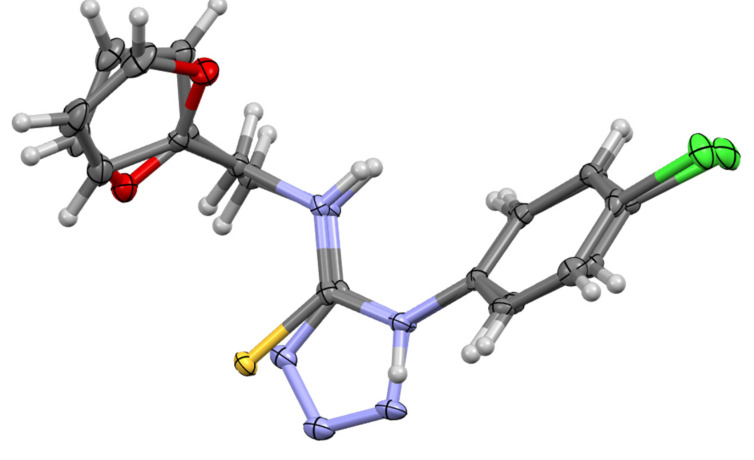
Superposition of **8a** and **8**.

Molecular structures of thiourea-based substrates **8a** and **8** are presented in Figure 3. Going from substrate to product twists the furan moiety with respect to chlorophenylthiourea/tetrazole skeleton (Figure 4). Compound **8a** is more bent, the angle between best planes calculated for furan and phenyl rings equals 65.7°, while for **8** it is 75.2°.

**Figure 5:**
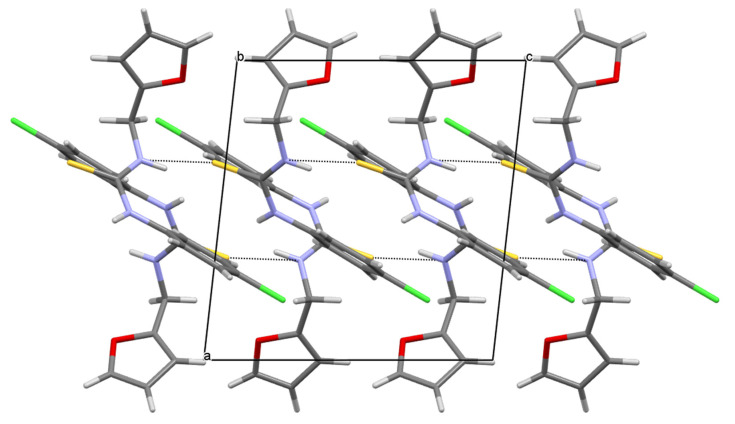
Intermolecular interactions in the crystal lattice along [010] direction for **8a**.

**Figure 6:**
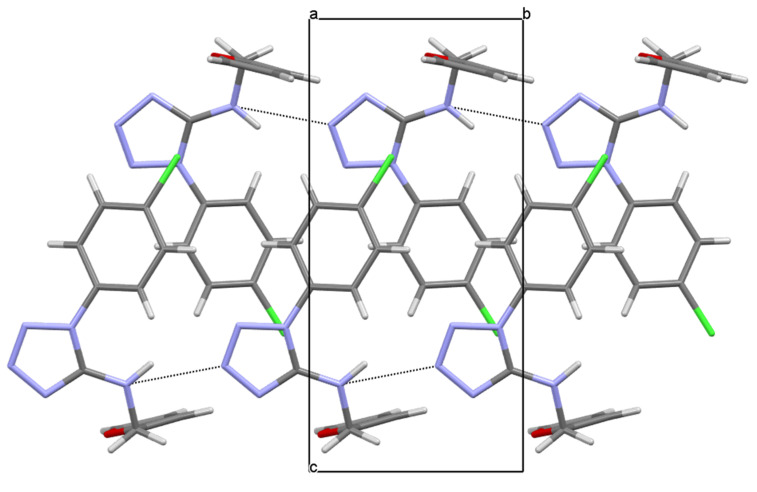
Intermolecular interactions in the crystal lattice along [100] direction for **8**.

The crystal structure of **8a** forms a chain along the y axis stabilized by a set of strong hydrogen bonds N1-H1 S1 (2.658Å, Figure 5), whereas **8** does so along x axis N1-H1 N4 (2.183Å, Figure 6). Additionally, there are intermolecular interactions C11-H11 N4 (2.689Å, see Figure S1, X-ray studies file, Supplementary Materials) for **8** and C-H S in the range of 2.783-2.853Å, C-H O in the range of 2.614-2.738Å for **8a** (see Figure S2, X-ray studies file, Supplementary Materials).

**Figure 7:**
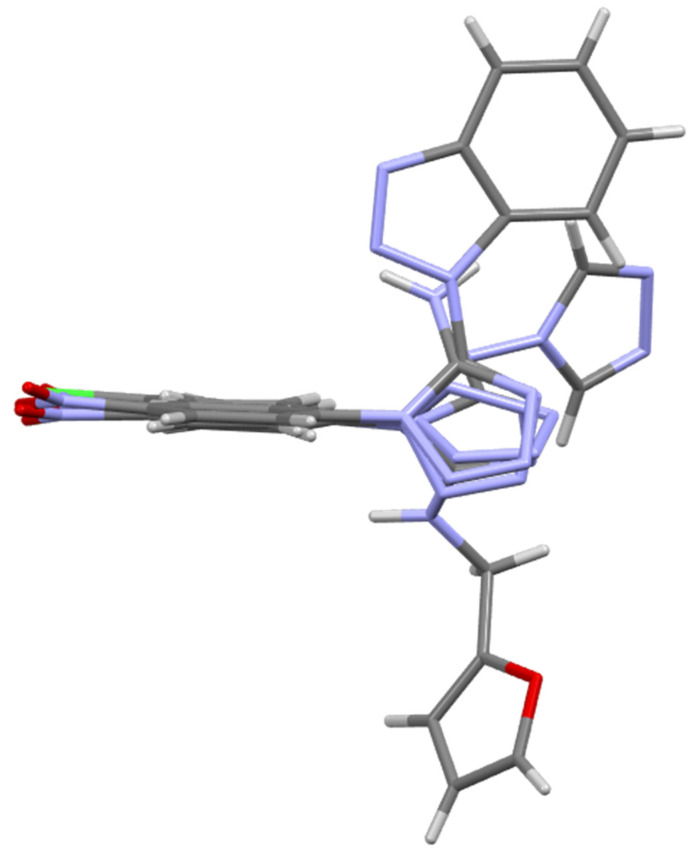
The comparison of four molecules taken from crystal structures—1-(4-chlorophenyl)-*N*-(furan-2-ylmethyl)-1*H*-tetrazol-5-amine **8**; JODHOA-1-(4-Nitrophenyl)-1*H*-tetrazol-5-amine [26]; YICNAA-1-(1-(4-Nitrophenyl)-1*H*-tetrazol-5-yl)-1H-1,2,3-benzotriazole [27]; JIKRAZ-(1-(4-nitrophenyl)-N-(4H-1,2,4-triazol-4-yl)-1H-tetrazol-5-aminido)-hexakis(aqua)-calcium 1-(4-nitrophenyl)-N-(4H-1,2,4-triazol-4-yl)-1H-tetrazol-5-aminide dehydrate [20].

To show structural diversities three compounds with similar phenyltetrazole substituted moieties have been found in Cambridge Structural Database [25]. The torsion angles between the tetrazole and phenyl ring are significantly different and equal for 8-140.62°; JODHOA-127.63°; YICNAA-89.10° and JIKRAZ-161.44° (Figure 7). This comparison shows how labile this fragment is and a small structural feature such as an additional ring or substituent may completely change the crystal structure. It may be important for the starting geometry in molecular docking studies, when real solid-state parameters are used instead of in silico data.

In a subsequent article, the comparisons between the two groups will be made in order to be able to draw unambiguous conclusions as to the change of the thiourea fragment to the tetrazole with a description of the physicochemical changes in the solid state. Firstly, a greater group of suitable thiourea-tetrazole derivative pairs must be collected.

### 2.4. Biological Studies

#### 2.4.1. In Vitro Antibacterial Activity Studies

Both disubstituted *N*-(furan-2-ylmethyl)-1*H*-tetrazol-5-amines studied by us recently [20] exerted excellent antimicrobial profiles. The 3-chloro-4-fluorophenyl derivative **Ref 2** was active against various standard bacterial strains at the range of 0.25-4 µg/mL, being also a strong inhibitor of the growth of clinical cocci (MICs of 2-16 µg/mL). Very similar results were also noted for its 3-chloro-4-methylphenyl Ref 1 analogue. Moreover, these tetrazol-5-amines were severalfold more active than the referential Ciprofloxacin.

Prompted by this, all obtained *N*-(furan-2-ylmethyl)-1*H*-tetrazol-5-amine derivatives **1**–**12** were tested in vitro against a number of bacteria, including representative standard Gram-positive and Gram-negative rods. Compounds were screened for their minimal inhibitory concentrations (MICs) [28]. The results revealed that half of investigated tetrazole derivatives exhibited moderate-to-weak antimicrobial properties, with MIC values ranging from 8 to 256 µg/mL (Table 2). Inactive compounds were not included in the table. The active compounds came mainly from the group of *para*-substituted **1**, **3** or disubstituted **3** tetrazoles, and the 2-fluorophenyl analogue **6** was the most promising. Its growth-inhibitory properties towards standard Staphylococci were in the range of 8–32 µg/mL, whereas it prevented the rise of some Gram-negative strains at the concentration of 16 µg/mL. Next, the tests performed against the hospital staphylococcal isolates panel completed the antimicrobial profile of derivative **6** (Table 3). It inhibited four hospital S. epidermidis rods growth, when applied at the amount of 4 µg/mL. The MICs towards the rest of studied Staphylococci were at the level of 8 µg/mL, except of *S. aureus 155/6* isolate (MIC = 16 µg/mL). However, the most susceptible to the presence of tested 2-fluorophenyltetrazole **6** was the *S. epidermidis T 5501 851/19* clinical strain, for which the MIC value was only 2 µg/mL. It is worth noting that this derivative’s growth inhibitory properties towards two clinical *S. epidermidis* strains was 8–16-fold times stronger than the referential chemotherapeutic. In addition, it exerted half of the Ciprofloxacin strength against other the staphylococcal KR 4047 825/19 isolate.

Results presented for **Ref 1** and **Ref 2** in the table above are not complete since the other strain collection was used in the previous project [20]. For the same reason we cannot compare the results for clinical strains. Collected clinical strains for current and previous antimicrobial evaluation are different. However, we want to point out that compound **Ref 1** and **Ref 2** possessed the highest antimicrobial potential against standard and clinical strains. We want to emphasize that derivative **6** can also be recognized as a leading compound because of a comparable level of antimicrobial activity.

#### 2.4.2. In Vitro Antimycobacterial Activity Studies

Inspired by biological properties observed for another series of 1*H*-tetrazol-5-amine derivatives [14], all new tetrazoles **1**–**12** were screened for their in vitro antitubercular properties against *M. tuberculosis* H_37_Rv strain and two “wild-type” mycobacteria isolated from tuberculosis patients—multidrug-resistant Spec. 210 (with resistance to p-aminosalicylic acid (PAS), isoniazid (INH), ethambutol (EMB) and rifampicin (RMP)) and Spec. 192, fully susceptible to established tuberculostatics (Table S2, Supplementary Materials). The compounds did not exert expected antimycobacterial activity (MIC values from 256 to 512 µg/mL).

#### 2.4.3. Cytotoxic Studies

To get information about cytotoxicity of new compounds **1**–**12** in mammalian cells, in vitro assays on three cancer cell lines were performed—HTB-140 (human melanoma), A549 (human epithelial lung carcinoma), Caco-2 (Caucasian colon adenocarcinoma), as well as on the normal HaCaT cell line were performed. The results (IC_50_ ˃ 100 µM) showed that studied tetrazoles did not influence the viability of these cells (Table S3, Supplementary Materials).

#### 2.4.4. Structure-activity Relationship Studies (SAR)

**Figure 8:**
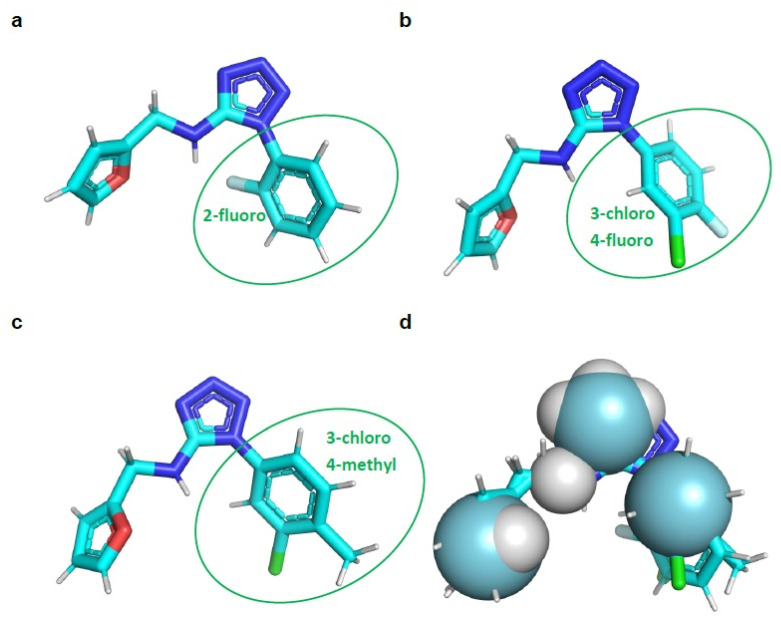
The leading compounds and their pharmacophore. Panels (**a**–**c**) show the conformations of the three leading molecules—compound **6** from this work and compound **Ref 2** and **Ref 1** from our previous work [20]. Panel (**d**) shows aligned structures of (**a**–**c**) together with pharmacophore units presented as spheres.

The molecule conformations were obtained using the ATB topology builder tool 3.0 quantum mechanical calculations combined with a knowledge-based approach [29]. Panel (d) shows aligned structures of (a), (b), and (c) together with pharmacophore units presented as spheres (Figure 8). The pharmacophore was detected via multiple alignments of the lead molecules using the PharmaGist tool [30]. We identified eight pharmacophore units—three aromatic, three acceptors in the tetrazole ring and two donors, oxygen in the furan ring, and the amine group’s nitrogen. Halogen substituents of the phenyl ring (marked by green circles) were not identified as pharmacophore units because of their different spatial positions, either as substituents in the ring and the ring’s different orientations that obviously may change in vivo. Thus, the role of fluorine or chlorine substituents in the antimicrobial activity is not evident in the pharmacophore structure analysis but may be important as indicated in our studies.

## 3. Materials and Methods

### 3.1. Apparatus, Materials, and Analysis

The reagents were supplied from Alfa Aesar (Haverhill, MA, USA) or Sigma Aldrich (Saint Louis, MO, USA). Organic solvents (acetonitrile, DMF, chloroform, and methanol) were supplied from POCh (Polskie Odczynniki Chemiczne, Gliwice, Poland). All chemicals were of analytical grade. Before use, dried DMF and acetonitrile were kept in crown cap bottles over anhydrous phosphorus pentoxide (Carl Roth, Karlsruhe, Germany).

The NMR spectra were recorded on a Bruker AVANCE DMX400 (Bruker, Billerica, MA, USA) spectrometer, operating at 500 MHz (^1^H NMR) and 125 MHz (^13^C NMR). The chemical shift values are expressed in ppm relative to TMS as an internal standard. Mass spectral ESI measurements were carried out on Waters ZQ Micro-mass instruments (Waters, Milford, MA, USA) with a quadrupol mass analyzer. The spectra were performed in the positive ion mode at a de-clustering potential of 40–60 V. The sample was previously separated on a UPLC column (C18) using the UPLC ACQUITYTM system by Waters connected with a DPA detector. Flash chromatography was performed on Merck silica gel 60 (200–400 mesh) using pure chloroform and chloroform/methanol (max. 19:1 vol) mixture as eluent. Analytical TLC was carried out on silica gel F254 (Merck) plates (0.25 mm thickness).

X-ray diffraction experiments were performed at 100(2) K for **8a** and **8**, respectively, on a Bruker D8 Venture Photon100 diffractometer (Bruker, Billerica, MA, USA) with a TRIUMPH monochromator and MoKα fine-focus sealed tube (λ = 0.71073 Å). The crystals were positioned 40 and 46 mm from the CCD camera for **8a** and **8**, respectively. A total of 901 and 689 frames were measured with exposure times of 30 min and 2,4 h for **8a** and **8**. Data were collected using the APEX2 program [31], integrated with the Bruker SAINT software package [32] and corrected for absorption effects using the multi-scan method (SADABS) [33]. The structures were solved and refined using the SHELX Software Package [34] with the atomic scattering factors taken from the International Tables [35]. The figures were generated with Mercury (ver. 2020.2.0) [36].

CCDC 2047281, 2047282 contains the supplementary crystallographic data for this paper. These data can be obtained free of charge via http://www.ccdc.cam.ac.uk/conts/retrieving.html.

### 3.2. Derivatives of N-(furan-2-ylmethyl)-1H-tetrazol-5-amine

Triethylamine (503 μL, 3.75 mmol, 1–3 drops) was added to a suspension of a corresponding thiourea substrate (1.25 mmol), sodium azide (244 mg, 3.75 mmol), and mercuric chloride (373 mg, 1.38 mmol) in 20 mL of dry DMF. The resulting suspension was stirred for 6 h at room temperature or until TLC indicated complete consumption of starting material. The suspension was filtered through a pad of celite, washing with CH_2_Cl_2_. The filtrate was diluted with water, and extracted with 3 × 15 mL of CH_2_Cl_2_. The combined organics were dried over MgSO_4_, filtered, and concentrated under reduced pressure. The resulting residue was purified by silica gel chromatography using pure chloroform initially as eluent, then increasingly adding methanol (max. 19:1 vol).

*1-(4-fluorophenyl)-N-(furan-2-ylmethyl)-1H-tetrazol-5-amine (****1****)*, Yield 83%. ^1^H NMR (500 MHz, CD_3_OD): δ 4.53 (s, 2H, CH_2_), 6.31–6.33 (m, 2H, Fur), 7.31–7.36 (m, 2H), 7.39–7.40 (m, 1H, Fur), 7.53–7.57 (m, 2H). ^13^C NMR (125 MHz, CD_3_OD): δ 41.6, 108.6 (Fur), 111.3 (Fur), 118.0 (d, ^2^*J* = 14.1 Hz, 2C), 128.4 (d, ^3^*J* = 5.5 Hz, 2C), 130.6 (d, ^4^*J* = 2.0 Hz), 143.4 (Fur), 152.9 (Fur), 156.5 (Tet), 164.5 (d, ^1^*J* = 148.4 Hz). HRMS (ESI) calc. for C_12_H_10_FN_5_O [M − H]^−^: 258,2444 found: 258,2441.

*1-(5-chloro-2-methylphenyl)-N-(furan-2-ylmethyl)-1H-tetrazol-5-amine(**2**)*, Yield 92%. ^1^H NMR (500 MHz, CD_3_OD): δ 2.02 (s, 3H), 4.51 (s, 2H, CH_2_), 6.29–6.30 (m, 1H, Fur), 6.33–6.34 (m, 1H, Fur), 7.40–7.42 (m, 2H), 7.44–7.46 (m, 1H, Fur), 7.52–7.54 (m, 2H). ^13^C NMR (125 MHz, CD_3_OD): δ 16.8, 41.4, 108.6 (Fur), 111.3 (Fur), 128.7, 132.2, 133.6, 133.9, 134.0, 136.3, 143.4 (Fur), 152.9 (Fur), 157.0 (Tet). HRMS (ESI) calc. for C_13_H_12_ClN_5_O [M − H]^−^: 288,7230 found: 288,7227.

*N-(furan-2-ylmethyl)-1-(4-iodophenyl)-1H-tetrazol-5-amine(**3**)*, Yield 89%. ^1^H NMR (500 MHz, CD_3_OD): δ 4.53 (s, 2H, CH_2_), 6.32-6.34 (m, 2H, Fur), 7.31–7.33 (m, 2H), 7.40–7.41 (m, 1H, Fur), 7.95–7.98 (m, 2H). ^13^C NMR (125 MHz, CD_3_OD): δ 41.6, 97.0, 108.7 (Fur), 111.4 (Fur), 127.4 (2C), 134.3, 140.4 (2C), 143.4 (Fur), 152.9 (Fur), 156.3 (Tet). HRMS (ESI) calc. for C_12_H_10_IN_5_O [M − H]^−^: 366,1505 found: 366,1503.

*1-(3-chlorophenyl)-N-(furan-2-ylmethyl)-1H-tetrazol-5-amine(**4**)*, Yield 79%. ^1^H NMR (500 MHz, CD_3_OD): δ 4.54 (s, 2H, CH_2_), 6.33 (s, 2H, Fur), 7.41 (s, 1H), 7.48–7.50 (m, 1H, Fur), 7.57–7.60 (m, 3H). ^13^C NMR (125 MHz, CD_3_OD): δ 41.6, 108.7 (Fur), 111.4 (Fur), 124.1, 125.8, 131.1, 132.4, 135.7, 136.5, 143.3 (Fur), 152.8 (Fur), 156.3 (Tet). HRMS (ESI) calc. for C_12_H_10_ClN_5_O [M − H]^−^: 274,6960 found: 274,6859.

*1-(3,4-dichlorophenyl)-N-(furan-2-ylmethyl)-1H-tetrazol-5-amine(**5**)*, Yield 88%. ^1^H NMR (500 MHz, CD_3_OD): δ 4.54 (s, 2H, CH_2_), 6.33 (d, *J* = 0.9 Hz, 2H, Fur), 7.41 (t, *J* = 0.9 Hz, 1H), 7.50 (dd, *J*_1_ = 1.5 Hz, *J*_2_ = 5.4 Hz, 1H, Fur), 7.75 (d, *J* = 5.1 Hz, 1H), 7.78 (d, *J* = 1.5 Hz, 1H). ^13^C NMR (125 MHz, CD_3_OD): δ 41.6, 108.8 (Fur), 111.4 (Fur), 125.5, 127.8, 132.7, 134.1, 134.7, 135.0, 143.5 (Fur), 152.7 (Fur), 156.3 (Tet). HRMS (ESI) calc. for C_12_H_9_Cl_2_N_5_O [M − H]^−^: 309,1380 found: 309,1378.

*1-(2-fluorophenyl)-N-(furan-2-ylmethyl)-1H-tetrazol-5-amine(**6**)*, Yield 90%. ^1^H NMR (500 MHz, CD_3_OD): δ 4.53 (s, 2H, CH_2_), 6.29-6.33 (m, 2H, Fur), 7.39–7.44 (m, 3H), 7.52–7.56 (m, 1H, Fur), 7.62–7.67 (m, 1H). ^13^C NMR (125 MHz, CD_3_OD): δ 41.5, 108.5 (Fur), 111.3 (Fur), 118.3 (d, ^2^*J* = 11.3 Hz), 121.9 (d, ^3^*J* = 7.6 Hz), 126.6 (d, ^4^*J* = 2.3 Hz), 130.0, 134.0 (d, ^3^*J* = 4.7 Hz), 143.4 (Fur), 152.9 (Fur), 157.2 (Tet), 158.4 (d, ^1^*J* = 151.0 Hz). HRMS (ESI) calc. for C_12_H_10_FN_5_O [M − H]^−^: 258,2444 found: 258,2441.

*1-(3-bromophenyl)-N-(furan-2-ylmethyl)-1H-tetrazol-5-amine(**7**),* Yield 85%. ^1^H NMR (500 MHz, CD_3_OD): δ 4.54 (s, 2H, CH_2_), 6.33 (s, 2H, Fur), 7.41 (s, 1H), 7.7.49−7.52 (m, 2H), 7.71-7.74 (m, 2H). ^13^C NMR (125 MHz, CD_3_OD): δ 41.6, 108.7 (Fur), 111.4 (Fur), 124.1, 124.5, 128.7, 132.6, 134.1, 135.8, 143.4 (Fur), 152.8 (Fur), 156.3 (Tet). HRMS (ESI) calc. for C_12_H_10_BrN_5_O [M − H]^−^: 319,1500 found: 319,1498.

*1-(4-chlorophenyl)-N-(furan-2-ylmethyl)-1H-tetrazol-5-amine(**8**),* Yield 81%. ^1^H NMR (500 MHz, CD_3_OD): δ 4.53 (s, 2H, CH_2_), 6.32–6.34 (m, 2H, Fur), 7.40–7.41 (m, 1H, Fur), 7.51–7.54 (m, 2H), 7.60–7.63 (m, 2H). ^13^C NMR (125 MHz, CD_3_OD): δ 41.6, 108.7 (Fur), 111.4 (Fur), 127.3 (2C), 131.2 (2C), 133.2, 136.8, 143.4 (Fur), 152.9 (Fur), 156.4 (Tet). HRMS (ESI) calc. for C_12_H_10_ClN_5_O [M − H]^−^: 274,6960 found: 274,6859.

*1-(3-fluorophenyl)-N-(furan-2-ylmethyl)-1H-tetrazol-5-amine(**9**),* Yield 89%. ^1^H NMR (500 MHz, CD_3_OD): δ 4.55 (s, 2H, CH_2_), 6.33−6.34 (m, 2H, Fur), 7.30–7.34 (m, 1H), 7.36–7.39 (m, 2H), 7.41–7.42 (m, 1H, Fur), 7.60–7.65 (m, 1H). ^13^C NMR (125 MHz, CD_3_OD): δ 41.6, 108.7 (Fur), 111.4 (Fur), 113.2 (d, ^2^*J* = 15.1 Hz), 117.8 (d, ^2^*J* = 12.7 Hz), 121.5 (d, ^4^*J* = 2.0 Hz), 132.8 (d, ^3^*J* = 5.4 Hz), 135.9 (d, ^3^*J* = 6.2 Hz), 143.4 (Fur), 152.9 (Fur), 156.3 (Tet), 164.3 (d, ^1^*J* = 147.9 Hz). HRMS (ESI) calc. for C_12_H_10_FN_5_O [M − H]^−^: 258,2444 found: 258,2441.

*1-(4-bromophenyl)-N-(furan-2-ylmethyl)-1H-tetrazol-5-amine(**10**),* Yield 83%. ^1^H NMR (500 MHz, CD_3_OD): δ 4.54 (s, 2H, CH_2_), 6.32–6.34 (m, 2H, Fur), 7.40–7.41 (m, 1H, Fur), 7.45–7.48 (m, 2H), 7.75–7.78 (m, 2H). ^13^C NMR (125 MHz, CD_3_OD): δ 41.6, 108.7 (Fur), 111.4 (Fur), 124.7, 127.35 (2C), 133.7, 134.3 (2C), 143.4 (Fur), 152.9 (Fur), 156.3 (Tet). HRMS (ESI) calc. for C_12_H_10_BrN_5_O [M − H]^−^: 319,1500 found: 319,1498.

*1-(2-chlorophenyl)-N-(furan-2-ylmethyl)-1H-tetrazol-5-amine(**11**),* Yield 80%. ^1^H NMR (500 MHz, CD_3_OD): δ 4.51 (s, 2H, CH_2_), 6.29–6.33 (m, 2H, Fur), 7.39–7.40 (m, 1H, Fur), 7.53–7.55 (m, 2H), 7.60–7.64 (m, 1H), 7.68–7.70 (m, 1H). ^13^C NMR (125 MHz, CD_3_OD): δ 41.5, 108.5 (Fur), 111.3 (Fur), 129.7, 130.9, 131.7, 132.0, 133.4, 133.7, 143.4 (Fur), 152.9 (Fur), 157.1 (Tet). HRMS (ESI) calc. for C_12_H_10_ClN_5_O [M − H]^−^: 274,6960 found: 274,6859.

*1-(2-bromophenyl)-N-(furan-2-ylmethyl)-1H-tetrazol-5-amine(**12**),* Yield 91%. ^1^H NMR (500 MHz, CD_3_OD): δ 4.51 (s, 2H, CH_2_), 6.30–6.33 (s, 2H, Fur), 7.39–7.40 (m, 1H, Fur), 7.51–7.56 (m, 2H), 7.58–7.61 (m, 1H), 7.85–7.87 (m, 1H). ^13^C NMR (125 MHz, CD_3_OD): δ 41.4, 108.5 (Fur), 111.3 (Fur), 123.1, 130.3, 131.1, 133.3, 133.9, 135.2, 143.3 (Fur), 152.9 (Fur), 157.0 (Tet). HRMS (ESI) calc. for C_12_H_10_BrN_5_O [M − H]^−^: 319,1500 found: 319,1498.

Copies of NMR spectra were added to supplementary part (NMR file).

### 3.3. Biological Assays

#### 3.3.1. Cytotoxic Studies (MTT Assay)

The cell viability was assessed by using an enzymatic conversion of 3-(4,5-dimethylthiazol-2-yl)-2,5-diphenyltetrazolium bromide salt (MTT) to insoluble formazan crystals by mitochondrial dehydrogenases, occurring in living cells. Cells were cultured in 96-well plates at a density of l × 10^4^ cells per well, and cultured to adhere for 24 h at 37 °C in a CO_2_ incubator. After 24 h of incubation, culture medium was replaced with a fresh medium, then cells were treated with various concentrations of the compounds and incubated for 72 h at 37 °C in a CO_2_ incubator. Untreated cells were used as the control. Subsequently, MTT solution (0,5 mg/mL in free-serum medium) was added, and samples were incubated for 4 h at 37 °C in a CO_2_ incubator. Then, the medium was aspirated, and formed formazan crystals were solubilized by adding an isopropanol-DMSO mixture (1:1 vol). The intensity of the dissolved crystals was measured using a UVM 340 reader (ASYS Hitech GmbH, Austria), at a wavelength of 570 nm. Cell viability was presented as a percent of MTT reduced in treated cells versus control cells (incubated without tested compounds). The relative MTT level (%) was calculated as [A]/[B]×100, where [A] is the absorbance of the test sample, and [B] is the absorbance of the control sample containing the untreated cells. The IC_50_ value was estimated using CalcuSyn version 1.0.

#### 3.3.2. Antimicrobial Activity

The antimicrobial assays were conducted using reference strains of bacteria derived from international microbe collections—American Type Culture Collection (ATCC) and National Collection of Type Culture (NCTC). Among the reference strains there were 4 Gram-positive isolates (*S. aureus*: NCTC 4163, ATCC 29213, *S. epidermidis* ATCC 12228 and *E. hirae* ATCC 10541) and 4 Gram-negative rods (*P. aeruginosa* ATCC 15442 and ATCC 27853, *E. coli* NCTC 8196 and ATCC 25922). The antimicrobial activity of compounds was expressed by minimum inhibitory concentration values (MICs), according to CLCI reference procedures with some modifications. MIC was tested by the two-fold serial microdilution method (in 96-well microtiter plates) on MH II liquid medium. The final inoculum of all studies bacteria was 106 CFU/mL (colony forming unit per ml). The stock solution of tested compounds was prepared in DMSO, and diluted in sterile medium (to a maximum of 1% of solvent content). The MIC value was the lowest concentration of the compound at which bacterial growth was no longer observed after 18 h of incubation at 35 °C. Clinical microorganisms used in this study were obtained from the collection of the Department of Pharmaceutical Microbiology, Medical University of Warsaw, Poland.

A detailed description related to all conducted biological studies including cell culture, suitable conditions, and methodology was presented in our previous papers [13,14,15,16,17,18,19,20,37].

## 4. Conclusions

Designed transition from thiourea substrates to appropriate *N*-(furan-2-ylmethyl)-1*H*-tetrazol-5-amines was successfully performed in the course of a single reaction. The synthesis was based on results from past studies and in silico conducted predictions. Within the obtained twelve derivatives, the 2-fluorophenyltetrazole **6** showed interesting activity against a panel of standard and clinical bacterial strains. In some cases of evaluation against clinical isolates, results were even better than for the reference Ciprofloxacin. For **8** and its corresponding substrate **8a**, single crystals were isolated and data gained related to physicochemical properties of the synthesized compounds. In the near future, for two groups, X-ray evaluation will be made to be able to draw unambiguous conclusions as to the change of the thiourea fragment with regards to the tetrazole one.

Biological evaluation results confirmed that none of tested derivatives were cytotoxic against normal HaCaT cell lines. None of synthesized compounds was recognized as a candidate for additional tests for antitubercular activity. In terms of seeking new antimicrobials, compounds **6** was found as an interesting leading structure. It inhibited four hospital *S. epidermidis* rods’ growth, when applied at the amount of 4 µg/mL. However, the most susceptible to the presence of tested 2-fluorophenyltetrazole **6** was the *S. epidermidis T 5501 851/19* clinical strain, for which the MIC value was only 2 µg/mL. In general, towards two clinical *S. epidermidis* strains it was 8–16-fold times stronger than the reference Ciprofloxacin.

Summarizing, *N*-(furan-2-ylmethyl)-1*H*-tetrazol-5-amines derivatives should be considered as promising in the field of antimicrobial action. The pharmacophore model was established. We identified eight pharmacophore units—three aromatic, three acceptors in the tetrazole ring and two donors, oxygen in the furan ring, and the amine group’s nitrogen. Phenyl substituents were not recognized as pharmacophore units. Further research will be performed on a broader group of compounds, especially those with fluorine-containing moieties attached to the phenyl ring.

## Figures and Tables

**Table 1:** The toxicological parameters of *N*-(furan-2-ylmethyl)-1*H*-tetrazol-5-amine derivatives estimated using pkCSM software.

Toxicological Test	Unit	1	2	3	4	5	6	7	8	9	10	11	12
AMES toxicity	(Yes/No)	No	No	No	No	No	No	No	No	No	No	No	No
Max. tolerated dose (human)	(log mg/kg/day)	0.056	0.14	0.016	0.038	0.074	0.045	0.031	0.039	0.055	0.032	0.028	0.021
hERG I inhibitor	(Yes/No)	No	No	No	No	No	No	No	No	No	No	No	No
hERG II inhibitor	(Yes/No)	No	No	No	No	No	No	No	No	No	No	No	No
Oral Rat Acute Toxicity (LD50)	(mol/kg)	2.069	2.211	2.148	2.145	2.254	2.052	2.154	2.151	2.064	2.16	2.133	2.141
Oral Rat Chronic Toxicity (LOAEL)	(log mg/kg_bw/day)	0.99	1.001	0.966	0.947	0.898	0.977	0.947	0.953	0.983	0.954	0.941	0.941
Hepatotoxicity	(Yes/No)	Yes	Yes	No	Yes	No	Yes	Yes	Yes	Yes	No	Yes	Yes
Skin Sensitisation	(Yes/No)	No	No	No	No	No	No	No	No	No	No	No	No
*T.Pyriformis* toxicity	(log ug/L)	0.34	0.341	0.342	0.34	0.346	0.337	0.341	0.342	0.338	0.343	0.34	0.34
Minnow toxicity	(log mM)	1.698	1.426	1.079	1.435	1.177	1.749	1.289	1.365	1.768	1.219	1.416	1.27

**Table 2:** Activity of compounds against standard bacteria strains, expressed by minimal inhibitory concentrations (µg/mL).

Strain	Compound								Ciprofloxacin [µg/mL]
	1	2	3	4	6	8	Ref 1	Ref 2	
*S. aureus NCTC 4163*	64	64	64	256	8	128	4	2	0.125
*S. aureus* ATCC 29213	64	64	64	256	8	128	-	-	0.5
*S. epidermidis* ATCC 12228	256	256	128	512	32	>	2	2	0,25
*E. hirae ATCC 10541*	128/64	64	128	512	16	>	8	4	2
*E. coli* ATCC 25922	128	128	128	>	16	>	-	-	0.015
*P. aeruginosa* ATCC 27853	>	>	>	>	>	>	4	2	0.25
*E. coli* NCTC 8196	128	128	128	512	512	>	-	-	0.015
*P. aeruginosa* ATCC 15442	>	>	512	>	>	>	32	64	0.125

**Table 3:** Activity of derivative **6** against clinical isolates, expressed by minimal inhibitory concentrations (µg/mL).

Strain	Compound 6 [µg/mL]	Ciprofloxacin [µg/mL]
*S. epidermidis KR 4047 825/19*	8	4
*S. epidermidis KR 4243 829/19*	4	0.015
*S. epidermidis KR 4268 830/19*	8	0.03
*S. epidermidis KR 4313 834/19*	4	64
*S. epidermidis KR 4358/2 840/19*	4	0.25
*S. epidermidis T 5253 845/19*	8	64
*S. epidermidis T 5399 848/19*	4	0.015
*S. epidermidis T 5501 851/19*	2	0.03
*S. aureus T5384/48*	8	0.2
*S. aureus 203/19*	8	0.1
*S. aureus T5403/51*	8	0.2
*S. aureus 176/13*	8	0.5
*S. aureus T5241/46*	8	0.1
*S. aureus 155/6*	16	0.1
*S. aureus T5591/68*	8	0.1
*S. aureus T5595/69*	8	0.1

## Data Availability

The data presented in this study are available on request from the corresponding author.

## Supplementary Materials

The following are available online. NMR file: ^1^H and ^13^C NMR, Table S2: *In vitro* evaluation of antitubercular properties, Table S3: Antiproliferative activity of tested compounds, X-ray studies file: Crystal data.

## Sample Availability

Samples of the compounds are not available from the authors.
