# Supplementary material for: Synthetic Transition from Thiourea-Based Compounds to Tetrazole Derivatives: Structure and Biological Evaluation of Synthesized New N-(Furan-2-ylmethyl)-1H-tetrazol-5-amine Derivatives
Source: Molecules. 2021 Jan 10;26(2):323. doi: 10.3390/molecules26020323 (PMC7827014; doi:10.3390/molecules26020323)

Supplementary material

Synthetic transition from thiourea-based compounds to tetrazole derivatives. Structure and biological evaluation of synthesized new *N*-(furan-2-ylmethyl)-1*H*-tetrazol-5-amine derivatives.

Daniel Szulczyk[a*], Anna Bielenica[a], Piotr Roszkowski[c], Michał A. Dobrowolski[c], Wioletta Olejarz[b], Sebastian Kmiecik[d], Małgorzata Podsiad[a] and Marta Struga[a].

[a]*Chair and Department of Biochemistry, Medical University of Warsaw, 02-097 Warszawa, Poland*

[b]*Department of Biochemistry and Pharmacogenomics, Faculty of Pharmacy, Medical University of Warsaw, 02-097 Warszawa, Poland*

[c]*Faculty of Chemistry, University of Warsaw, Pasteura 1, 02-093 Warszawa, Poland*

[d]*Biological and Chemical Research Centre, Faculty of Chemistry, University of Warsaw, 02-089 Warsaw, Poland*

^{1}H and ^{13}C NMR;

Compound 1

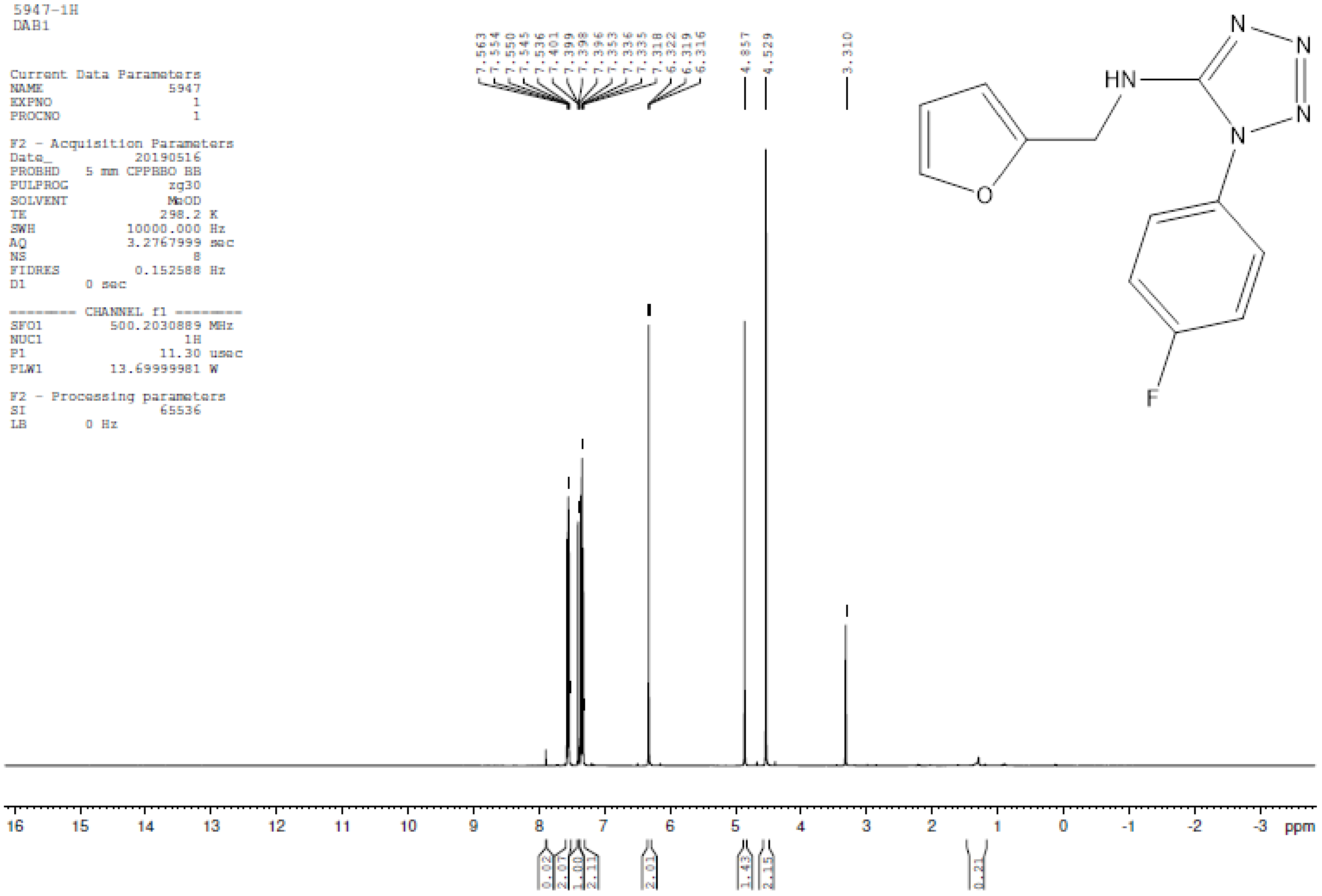
5947-1H
DAB1
Current Data Parameters
NAME 5947
EXPNO 1
PROCNO 1
F2 - Acquisition Parameters
Date_ 20190516
PROBHD 5 mm CPPBBO BB
PULPROG zg30
SOLVENT MeOD
TE 298.2 K
SWH 10000.000 Hz
AQ 3.2767999 sec
NS 8
FIDRES 0.152588 Hz
D1 0 sec
======== CHANNEL f1 ========
SFO1 500.2030889 MHz
NUC1 1H
P1 11.30 usec
PLW1 13.69999981 W
F2 - Processing parameters
SI 65536
LB 0 Hz
7.563
7.554
7.550
7.545
7.536
7.401
7.399
7.398
7.396
7.353
7.336
7.335
7.318
6.322
6.319
6.316
4.857
4.529
3.310
HN
N
N
N
N
O
F
16 15 14 13 12 11 10 9 8 7 6 5 4 3 2 1 0 -1 -2 -3 ppm
0.02
2.07
1.00
2.11
2.01
1.43
2.15
0.21

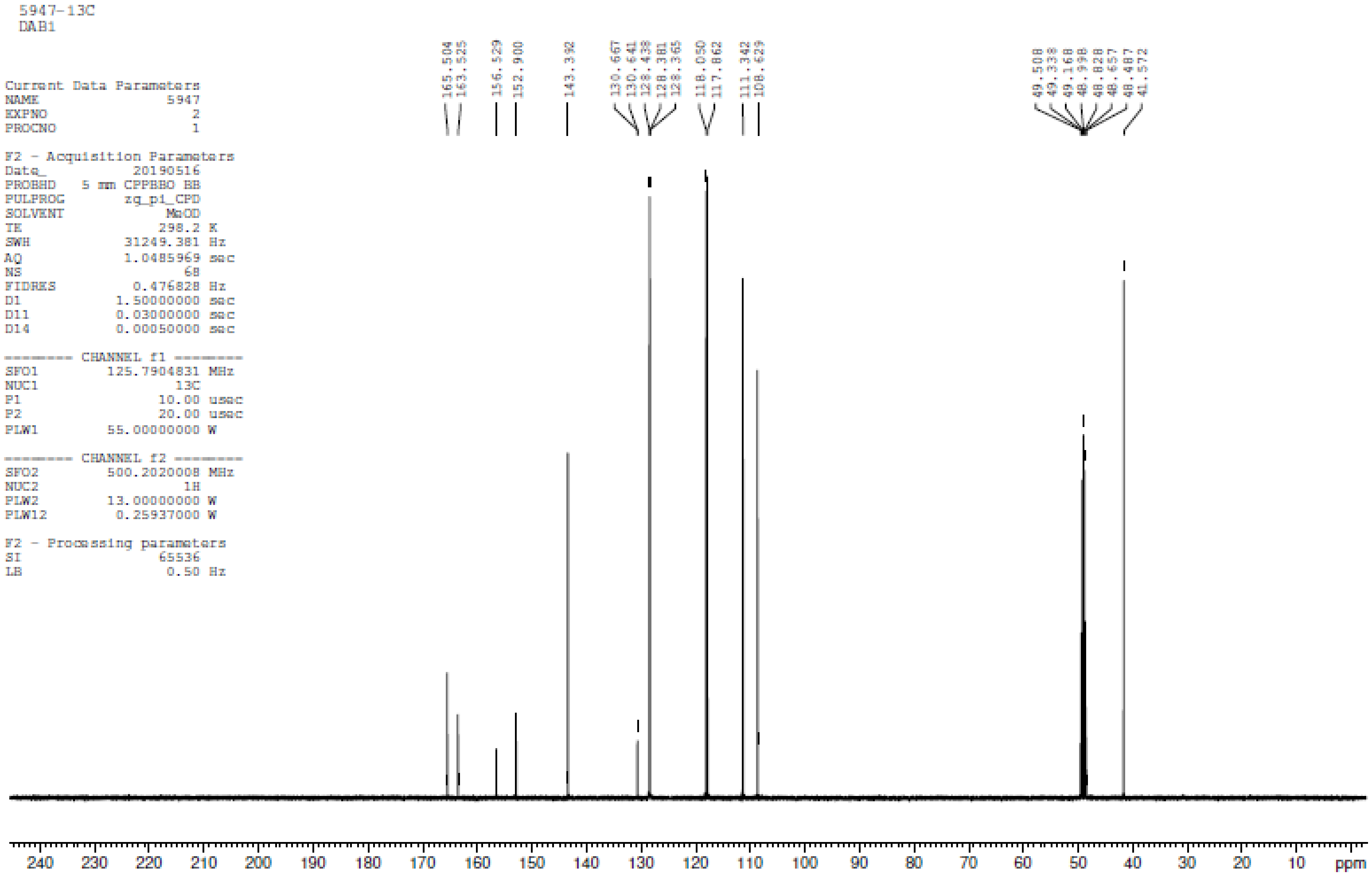
5947-13C
DAB1
Current Data Parameters
NAME 5947
EXPNO 2
PROCNO 1
F2 - Acquisition Parameters
Date_ 20190516
PROBHD 5 mm CPPBBO BB
PULPROG zg_pi_CPD
SOLVENT MeOD
TE 298.2 K
SWH 31249.381 Hz
AQ 1.0485969 sec
NS 68
FIDRES 0.476828 Hz
D1 1.50000000 sec
D11 0.03000000 sec
D14 0.00050000 sec
======== CHANNEL f1 ========
SFO1 125.7904831 MHz
NUC1 13C
P1 10.00 usec
P2 20.00 usec
PLW1 55.00000000 W
======== CHANNEL f2 ========
SFO2 500.2020008 MHz
NUC2 1H
PLW2 13.00000000 W
PLW12 0.25937000 W
F2 - Processing parameters
SI 65536
LB 0.50 Hz
165.504
163.525
156.529
152.900
143.392
130.667
130.641
128.438
128.381
128.365
118.050
117.862
111.342
108.629
49.508
49.338
49.168
48.998
48.828
48.657
48.487
41.572
240 230 220 210 200 190 180 170 160 150 140 130 120 110 100 90 80 70 60 50 40 30 20 10 ppm

Compound 2

5948-1H
DAB2

Current Data Parameters
NAME 5948
EXPNO 1
PROCNO 1

F2 - Acquisition Parameters
Date_ 20190516
PROBHD 5 mm CPPBBO BB
PULPROG zg30
SOLVENT MeOD
TE 298.2 K
SWH 10000.000 Hz
AQ 3.2767999 sec
NS 8
FIDRES 0.152588 Hz
D1 0 sec

======== CHANNEL f1 ========
SFO1 500.2030889 MHz
NUC1 1H
P1 11.30 usec
PLW1 13.69999981 W

F2 - Processing parameters
SI 65536
LB 0 Hz

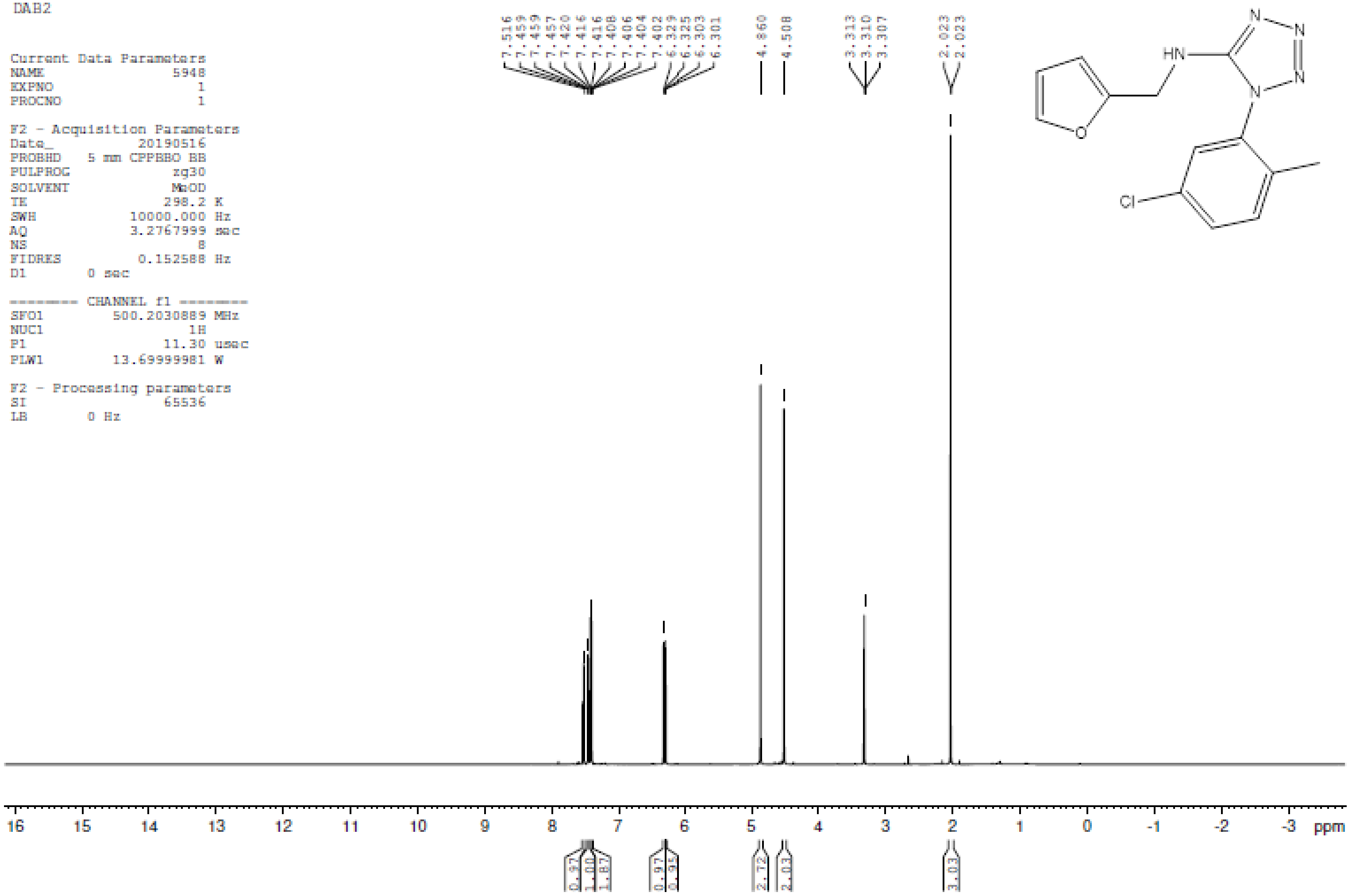

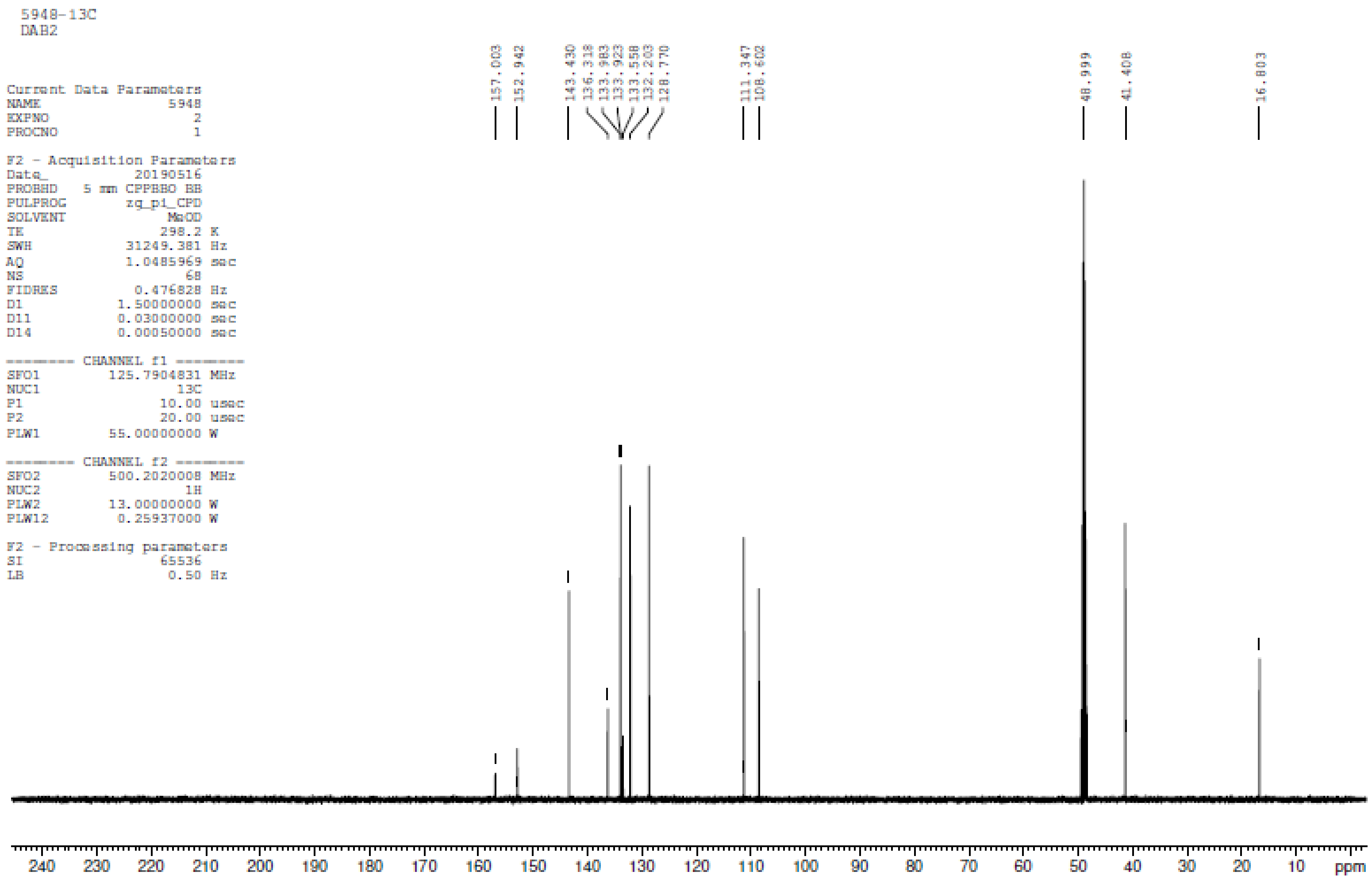
5948-13C
DAB2
Current Data Parameters
NAME 5948
EXPNO 2
PROCNO 1
F2 - Acquisition Parameters
Date_ 20190516
PROBHD 5 mm CPPBBO BB
PULPROG zg_pi_CPD
SOLVENT MeOD
TE 298.2 K
SWH 31249.381 Hz
AQ 1.0485969 sec
NS 68
FIDRES 0.476828 Hz
D1 1.50000000 sec
D11 0.03000000 sec
D14 0.00050000 sec
======== CHANNEL f1 ========
SFO1 125.7904831 MHz
NUC1 13C
P1 10.00 usec
P2 20.00 usec
PLW1 55.00000000 W
======== CHANNEL f2 ========
SFO2 500.2020008 MHz
NUC2 1H
PLW2 13.00000000 W
PLW12 0.25937000 W
F2 - Processing parameters
SI 65536
LB 0.50 Hz
157.003
152.942
143.430
136.318
133.983
133.923
133.558
132.203
128.770
111.347
108.602
48.999
41.408
16.803
240
230
220
210
200
190
180
170
160
150
140
130
120
110
100
90
80
70
60
50
40
30
20
10
ppm

Compound 3

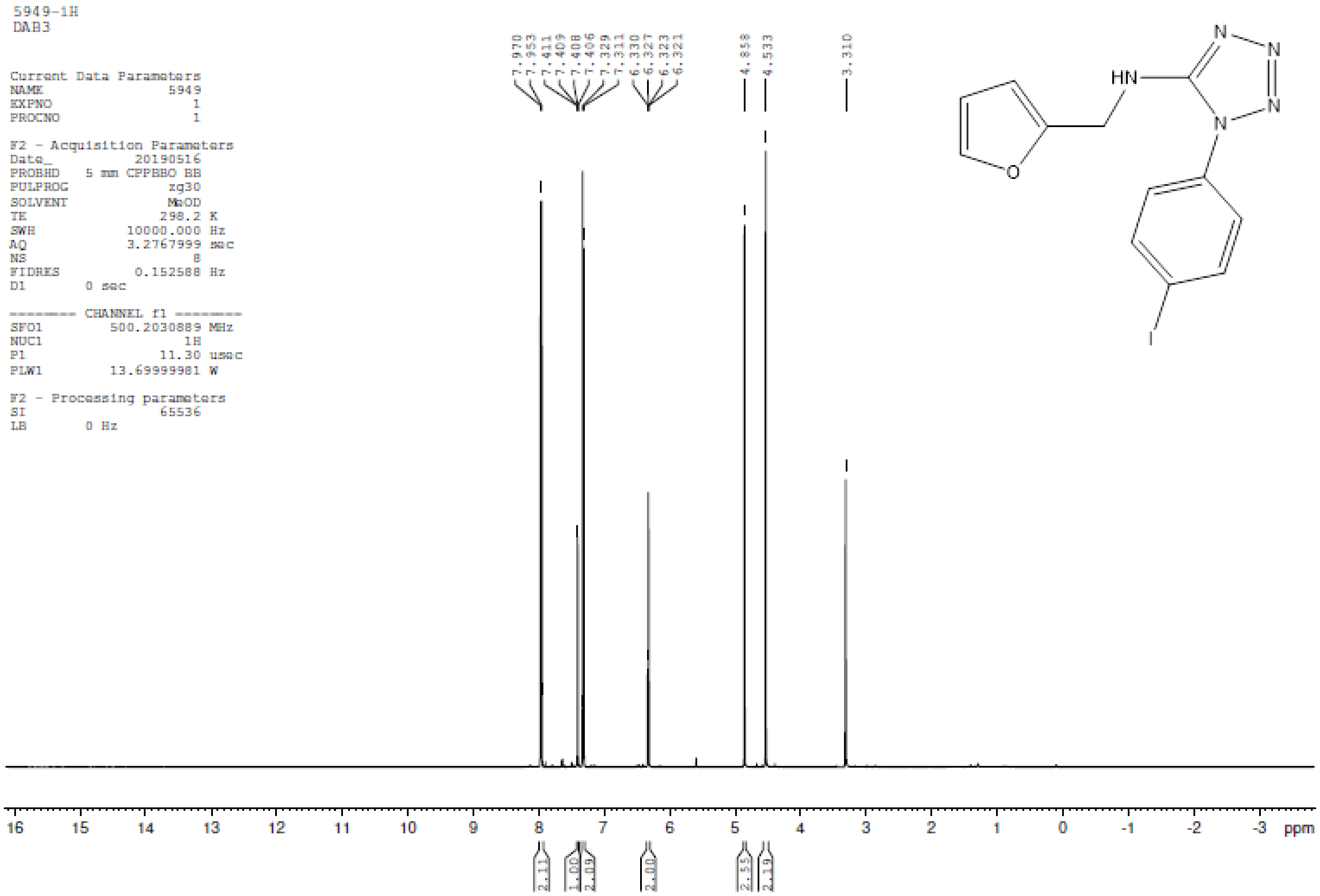
5949-1H
DAB3
Current Data Parameters
NAME 5949
EXPNO 1
PROCNO 1
F2 - Acquisition Parameters
Date_ 20190516
PROBHD 5 mm CPPBBO BB
PULPROG zg30
SOLVENT MeOD
TE 298.2 K
SWH 10000.000 Hz
AQ 3.2767999 sec
NS 8
FIDRES 0.152588 Hz
D1 0 sec
======== CHANNEL f1 ========
SFO1 500.2030889 MHz
NUC1 1H
P1 11.30 usec
PLW1 13.69999981 W
F2 - Processing parameters
SI 65536
LB 0 Hz
7.970
7.953
7.411
7.409
7.408
7.406
7.329
7.311
6.330
6.327
6.323
6.321
4.858
4.533
3.310
HN
N
N
N
N
O
I
16 15 14 13 12 11 10 9 8 7 6 5 4 3 2 1 0 -1 -2 -3 ppm
2.11
1.00
2.09
2.00
2.55
2.19

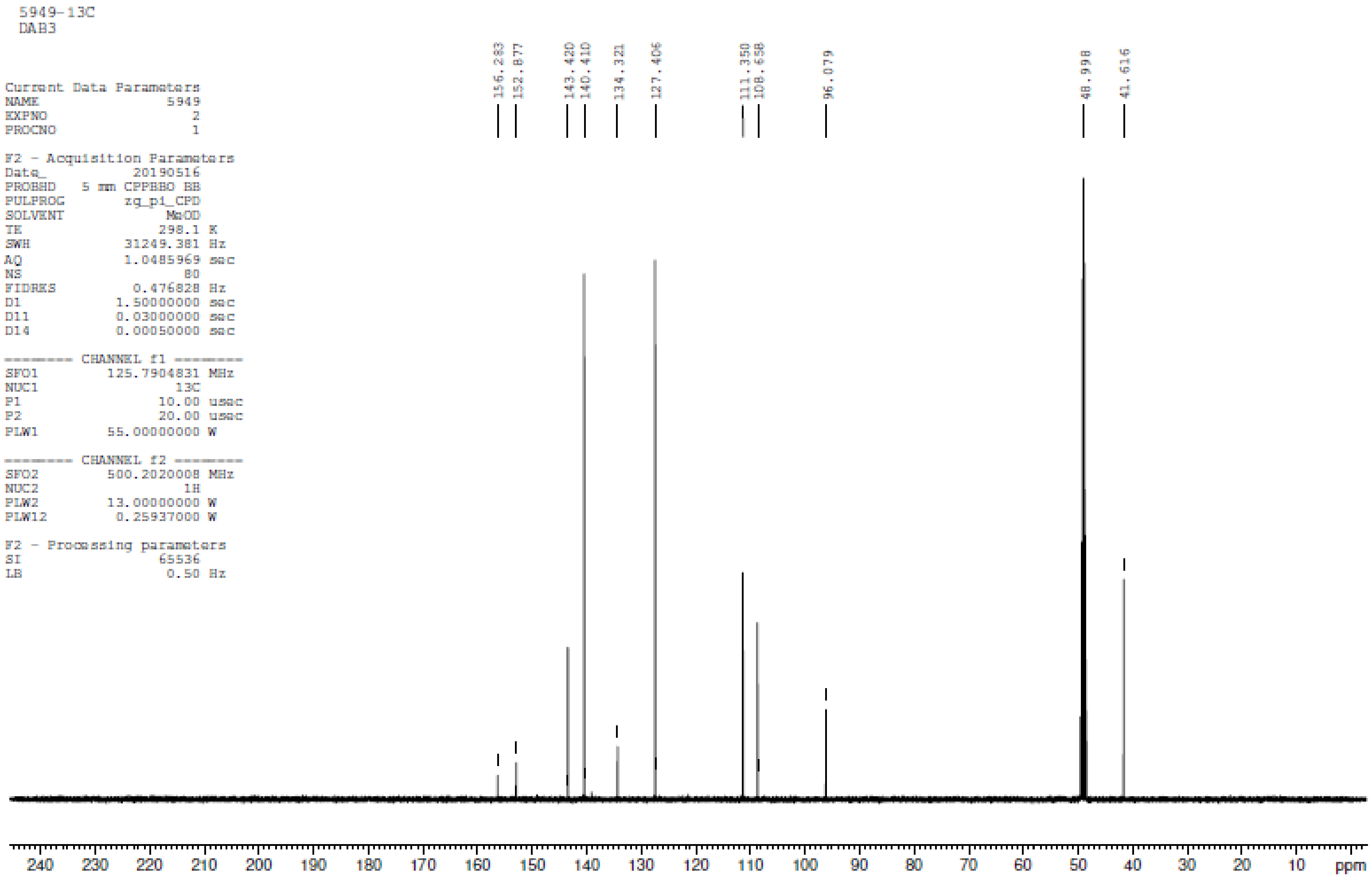
5949-13C
DAB3
Current Data Parameters
NAME 5949
EXPNO 2
PROCNO 1
F2 - Acquisition Parameters
Date_ 20190516
PROBHD 5 mm CPPBBO BB
PULPROG zg_pi_CPD
SOLVENT MeOD
TE 298.1 K
SWH 31249.381 Hz
AQ 1.0485969 sec
NS 80
FIDRES 0.476828 Hz
D1 1.50000000 sec
D11 0.03000000 sec
D14 0.00050000 sec
======== CHANNEL f1 ========
SFO1 125.7904831 MHz
NUC1 13C
P1 10.00 usec
P2 20.00 usec
PLW1 55.00000000 W
======== CHANNEL f2 ========
SFO2 500.2020008 MHz
NUC2 1H
PLW2 13.00000000 W
PLW12 0.25937000 W
F2 - Processing parameters
SI 65536
LB 0.50 Hz
156.283
152.877
143.420
140.410
134.321
127.406
111.350
108.658
96.079
48.998
41.616
240 230 220 210 200 190 180 170 160 150 140 130 120 110 100 90 80 70 60 50 40 30 20 10 ppm

Compound 4

5950-1H
DAB4

Current Data Parameters
NAME 5950
EXPNO 1
PROCNO 1

F2 - Acquisition Parameters
Date_ 20190516
PROBHD 5 mm CPPBBO BB
PULPROG zg30
SOLVENT MeOD
TE 298.2 K
SWH 10000.000 Hz
AQ 3.2767999 sec
NS 8
FIDRES 0.152588 Hz
D1 0 sec

======== CHANNEL f1 ========
SFO1 500.2030889 MHz
NUC1 1H
P1 11.30 usec
PLW1 13.69999981 W

F2 - Processing parameters
SI 65536
LB 0 Hz

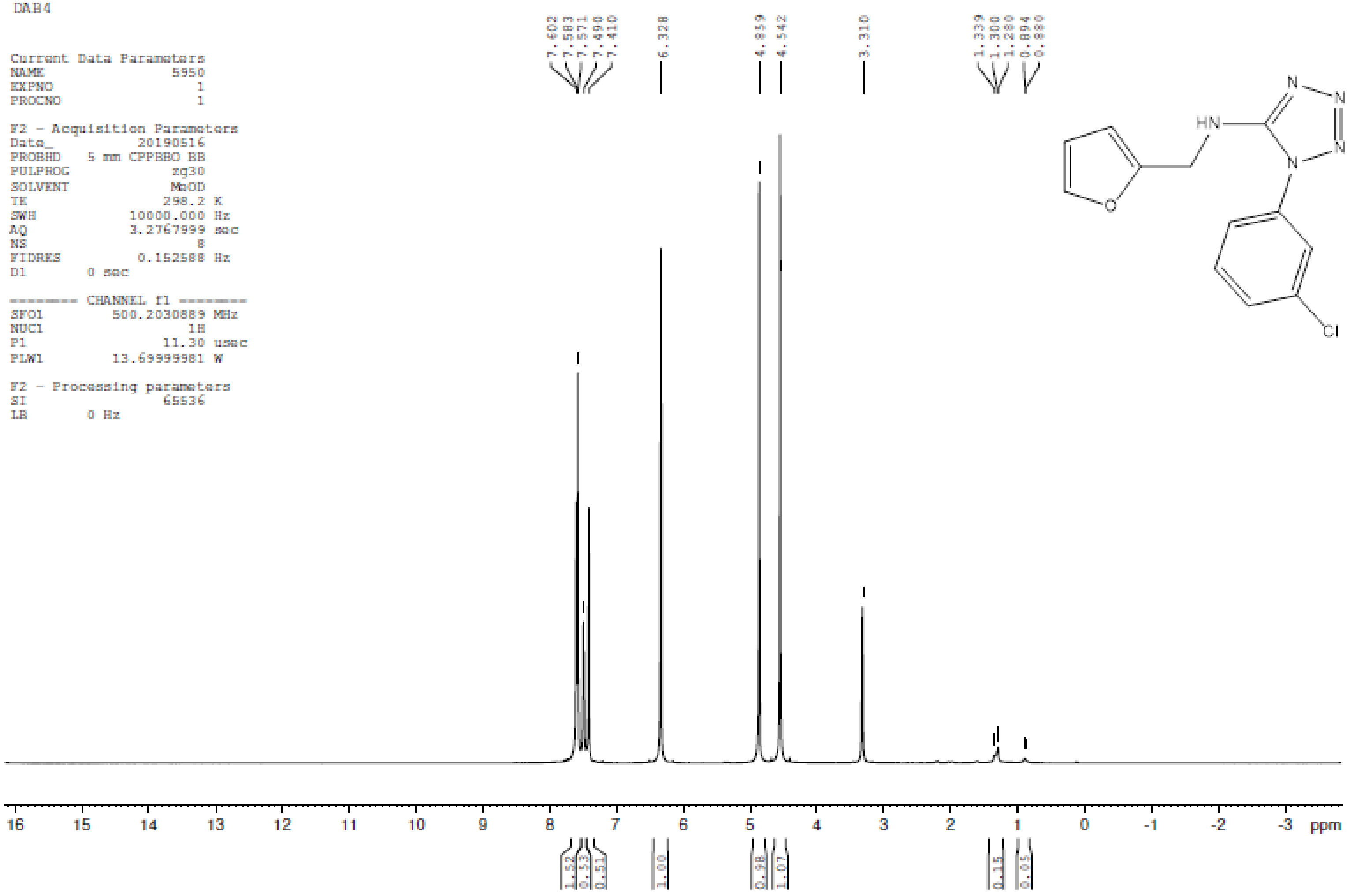

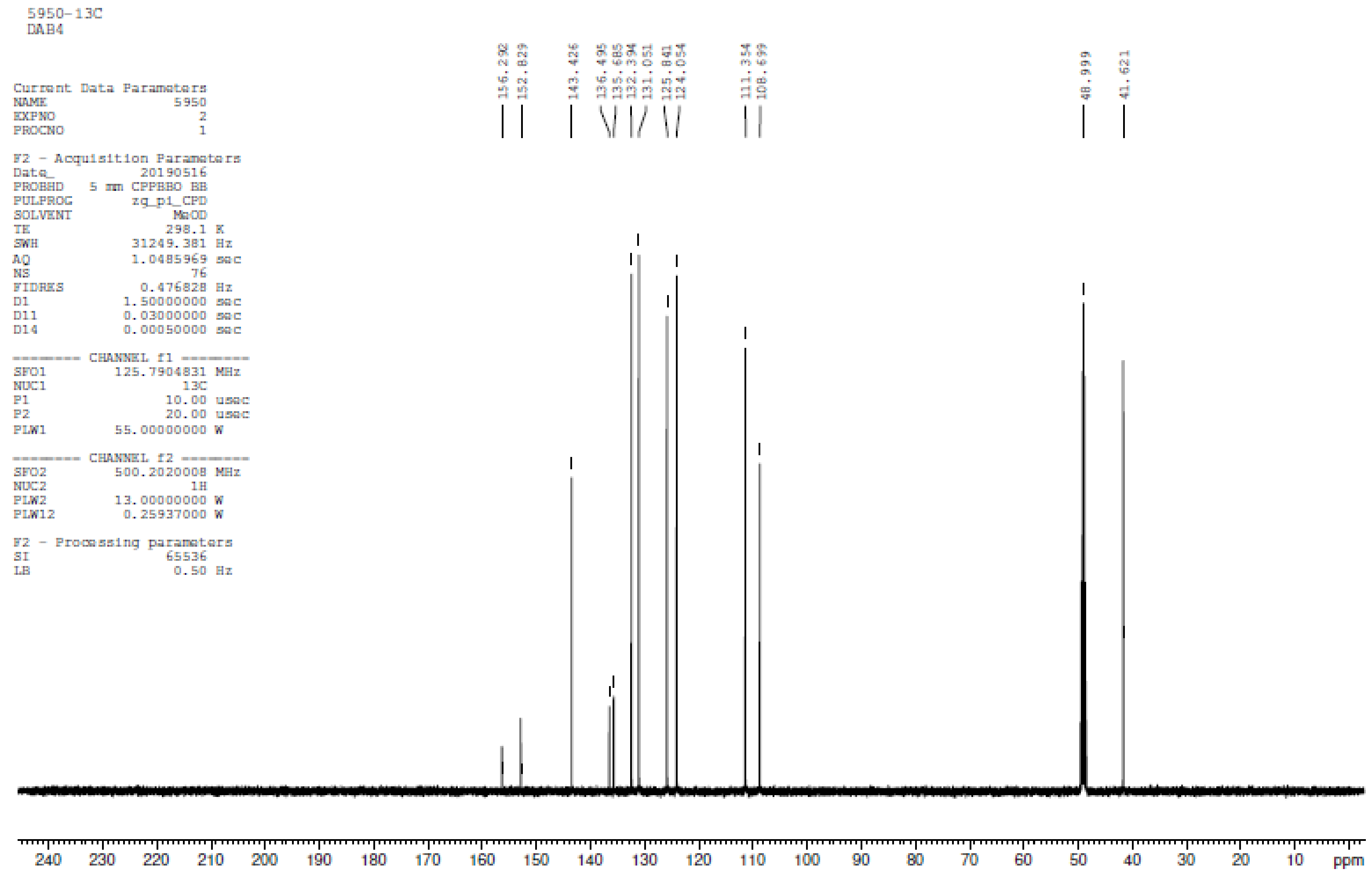
5950-13C
DAB4
156.292
152.829
143.426
136.495
135.685
132.394
131.051
125.841
124.054
111.354
108.699
48.999
41.621
Current Data Parameters
NAME 5950
EXPNO 2
PROCNO 1
F2 - Acquisition Parameters
Date_ 20190516
PROBHD 5 mm CPPBBO BB
PULPROG zg_pi_CPD
SOLVENT MeOD
TE 298.1 K
SWH 31249.381 Hz
AQ 1.0485969 sec
NS 76
FIDRES 0.476828 Hz
D1 1.50000000 sec
D11 0.03000000 sec
D14 0.00050000 sec
======== CHANNEL f1 ========
SFO1 125.7904831 MHz
NUC1 13C
P1 10.00 usec
P2 20.00 usec
PLW1 55.00000000 W
======== CHANNEL f2 ========
SFO2 500.2020008 MHz
NUC2 1H
PLW2 13.00000000 W
PLW12 0.25937000 W
F2 - Processing parameters
SI 65536
LB 0.50 Hz
240 230 220 210 200 190 180 170 160 150 140 130 120 110 100 90 80 70 60 50 40 30 20 10 ppm

Compound 5

5951-1H
DAB5

Current Data Parameters
NAME 5951
EXPNO 1
PROCNO 1

F2 - Acquisition Parameters
Date_ 20190517
PROBHD 5 mm CPPBBO BB
PULPROG zg30
SOLVENT MeOD
TE 298.2 K
SWH 10000.000 Hz
AQ 3.2767999 sec
NS 8
FIDRES 0.152588 Hz
D1 0 sec

======== CHANNEL f1 ========
SFO1 500.2030889 MHz
NUC1 1H
P1 11.30 usec
PLW1 13.69999981 W

F2 - Processing parameters
SI 65536
LB 0 Hz

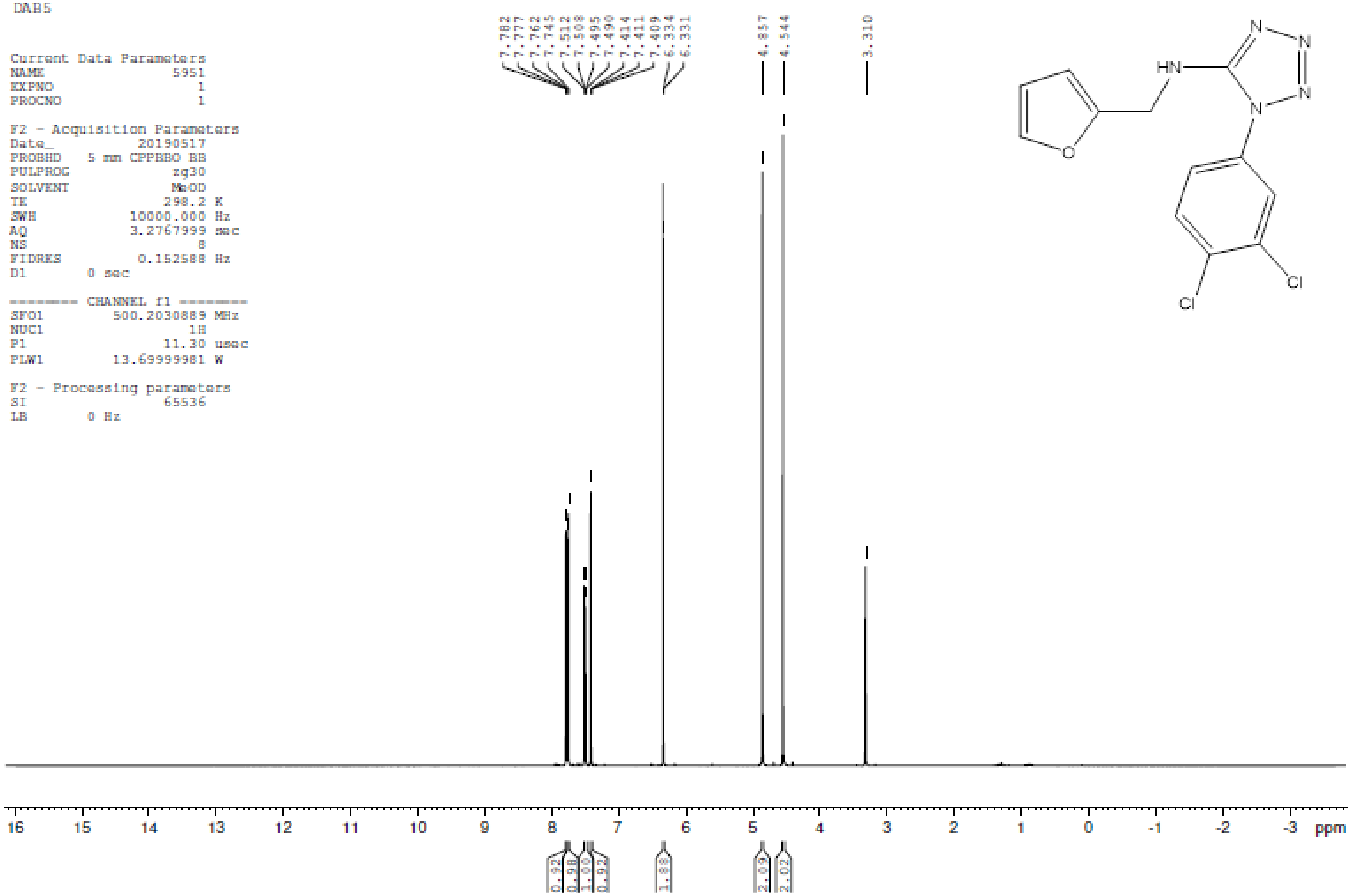

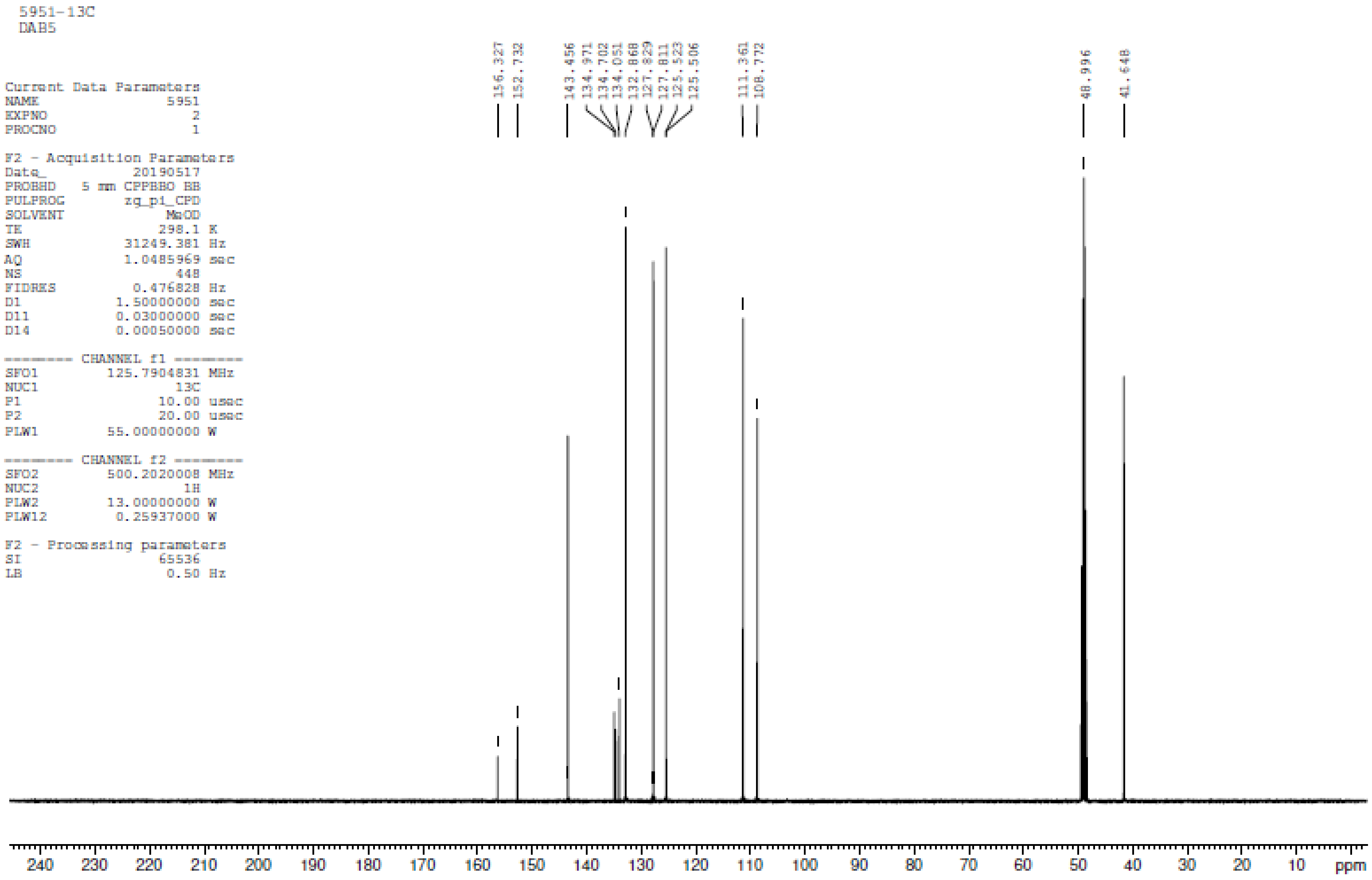
5951-13C
DAB5
Current Data Parameters
NAME 5951
EXPNO 2
PROCNO 1
F2 - Acquisition Parameters
Date_ 20190517
PROBHD 5 mm CPPBBO BB
PULPROG zg_pi_CPD
SOLVENT MeOD
TE 298.1 K
SWH 31249.381 Hz
AQ 1.0485969 sec
NS 448
FIDRES 0.476828 Hz
D1 1.50000000 sec
D11 0.03000000 sec
D14 0.00050000 sec
======== CHANNEL f1 ========
SFO1 125.7904831 MHz
NUC1 13C
P1 10.00 usec
P2 20.00 usec
PLW1 55.00000000 W
======== CHANNEL f2 ========
SFO2 500.2020008 MHz
NUC2 1H
PLW2 13.00000000 W
PLW12 0.25937000 W
F2 - Processing parameters
SI 65536
LB 0.50 Hz
156.327
152.732
143.456
134.971
134.702
134.051
132.868
127.829
127.811
125.523
125.506
111.361
108.772
48.996
41.648
240 230 220 210 200 190 180 170 160 150 140 130 120 110 100 90 80 70 60 50 40 30 20 10 ppm

Compound 6

5952-1H
DAB6

Current Data Parameters
NAME 5952
EXPNO 1
PROCNO 1

F2 - Acquisition Parameters
Date_ 20190517
PROBHD 5 mm CPPBBO BB
PULPROG zg30
SOLVENT DMSO
TE 298.1 K
SWH 10000.000 Hz
AQ 3.2767999 sec
NS 8
FIDRES 0.152588 Hz
D1 0 sec

======== CHANNEL f1 ========
SFO1 500.2030889 MHz
NUC1 1H
P1 11.30 usec
PLW1 13.69999981 W

F2 - Processing parameters
SI 65536
LB 0 Hz

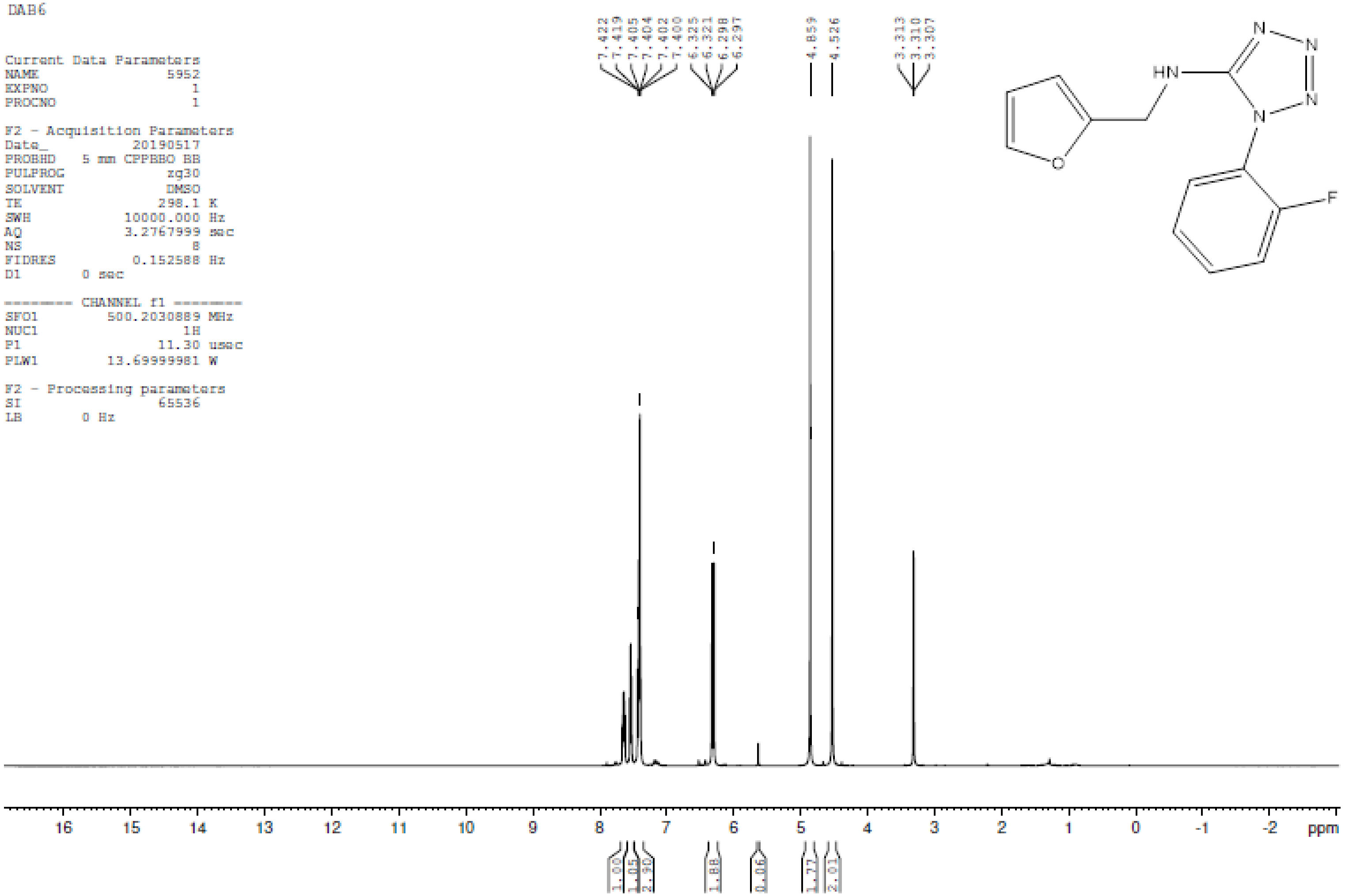

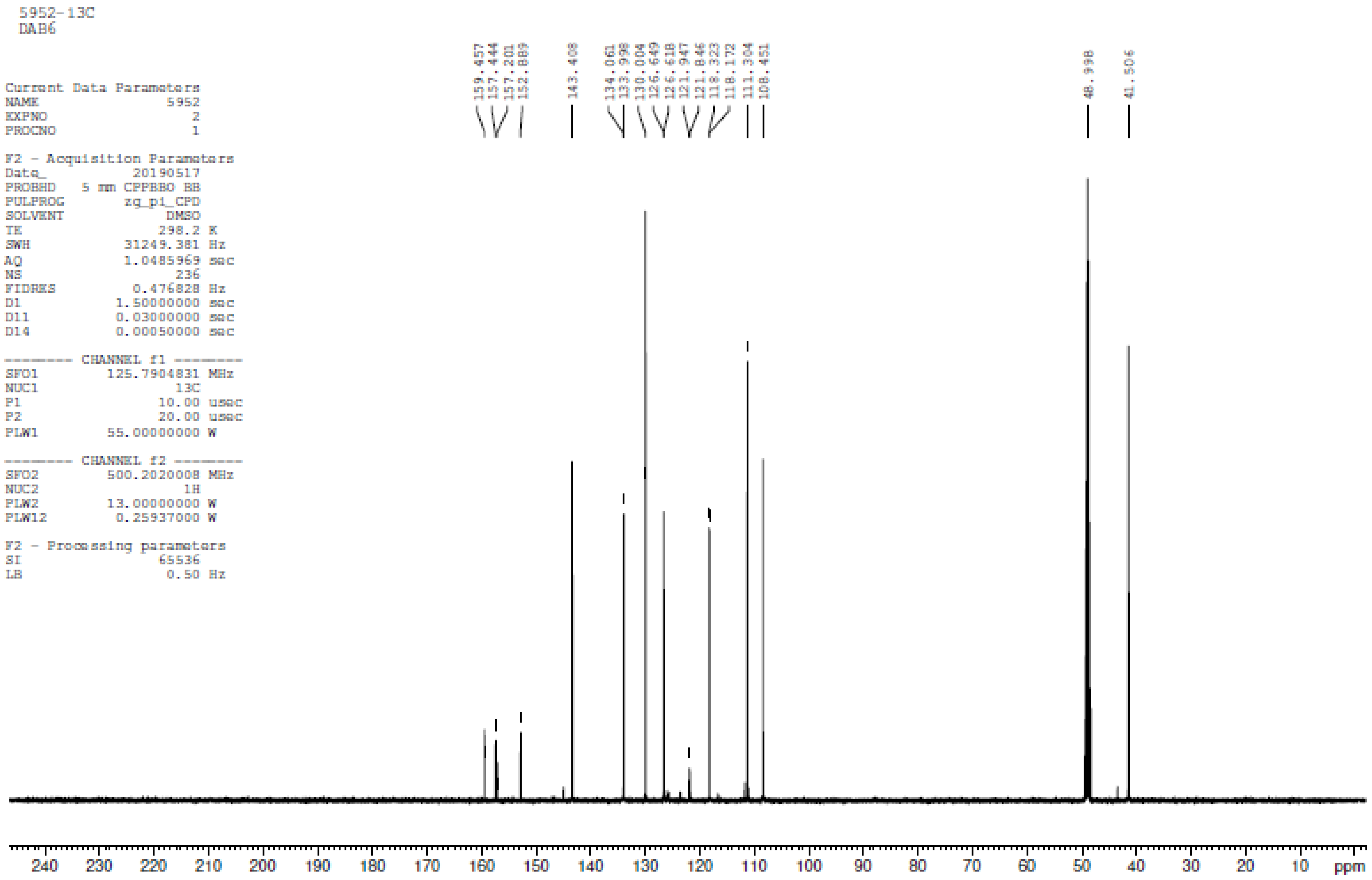
5952-13C
DAB6
Current Data Parameters
NAME 5952
EXPNO 2
PROCNO 1
F2 - Acquisition Parameters
Date_ 20190517
PROBHD 5 mm CPPBBO BB
PULPROG zg_pi_CPD
SOLVENT DMSO
TE 298.2 K
SWH 31249.381 Hz
AQ 1.0485969 sec
NS 236
FIDRES 0.476828 Hz
D1 1.50000000 sec
D11 0.03000000 sec
D14 0.00050000 sec
======== CHANNEL f1 ========
SFO1 125.7904831 MHz
NUC1 13C
P1 10.00 usec
P2 20.00 usec
PLW1 55.00000000 W
======== CHANNEL f2 ========
SFO2 500.2020008 MHz
NUC2 1H
PLW2 13.00000000 W
PLW12 0.25937000 W
F2 - Processing parameters
SI 65536
LB 0.50 Hz
159.457
157.444
157.201
152.889
143.408
134.061
133.998
130.004
126.649
126.618
121.947
121.846
118.323
118.172
111.304
108.451
48.998
41.506
240 230 220 210 200 190 180 170 160 150 140 130 120 110 100 90 80 70 60 50 40 30 20 10 ppm

Compound 7

5953-1H
DAB7

Current Data Parameters
NAME 5953
EXPNO 1
PROCNO 1

F2 - Acquisition Parameters
Date_ 20190517
PROBHD 5 mm CPPBBO BB
PULPROG zg30
SOLVENT DMSO
TE 298.2 K
SWH 10000.000 Hz
AQ 3.2767999 sec
NS 8
FIDRES 0.152588 Hz
D1 0 sec

======== CHANNEL f1 ========
SFO1 500.2030889 MHz
NUC1 1H
P1 11.30 usec
PLW1 13.69999981 W

F2 - Processing parameters
SI 65536
LB 0 Hz

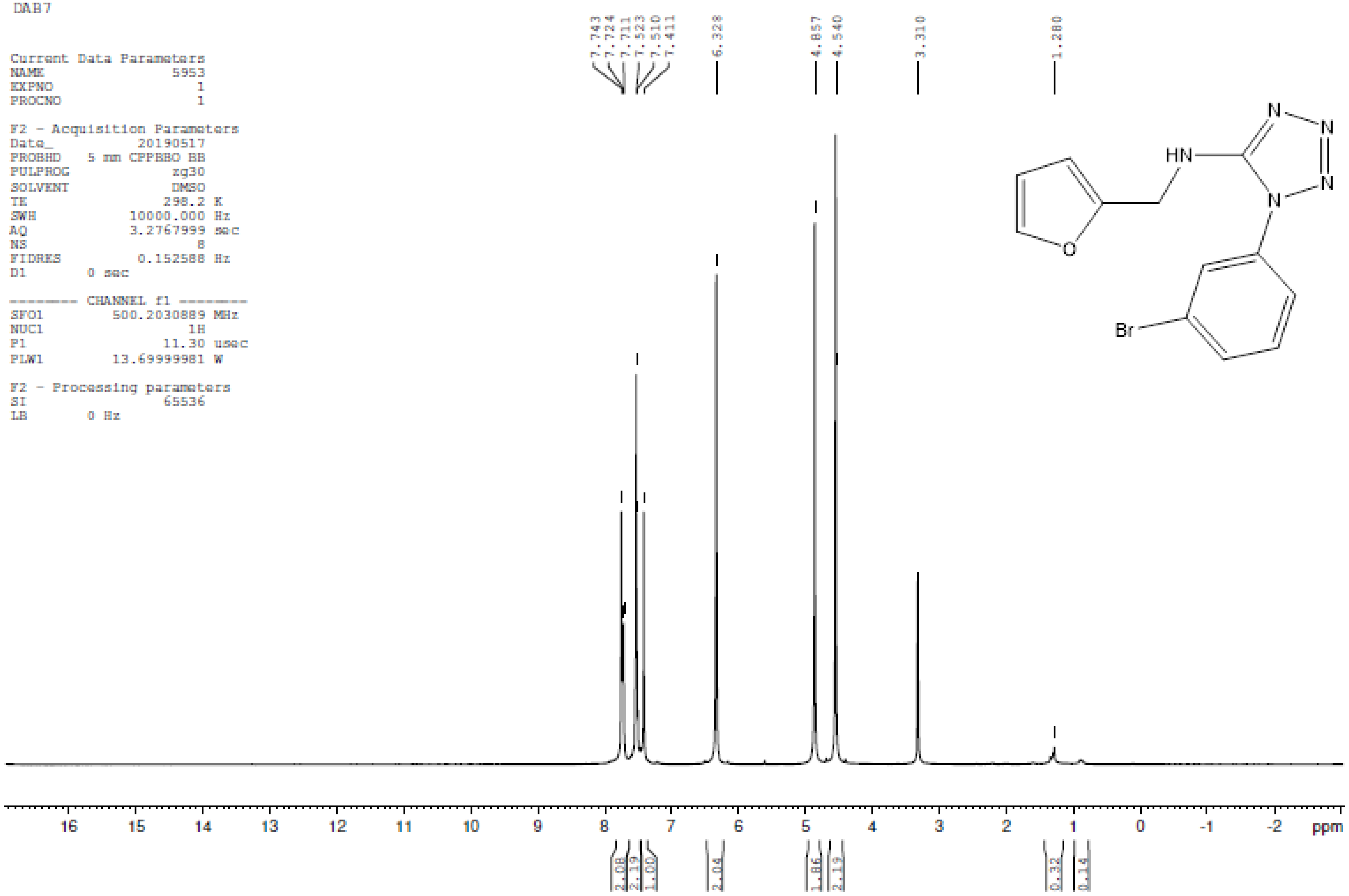

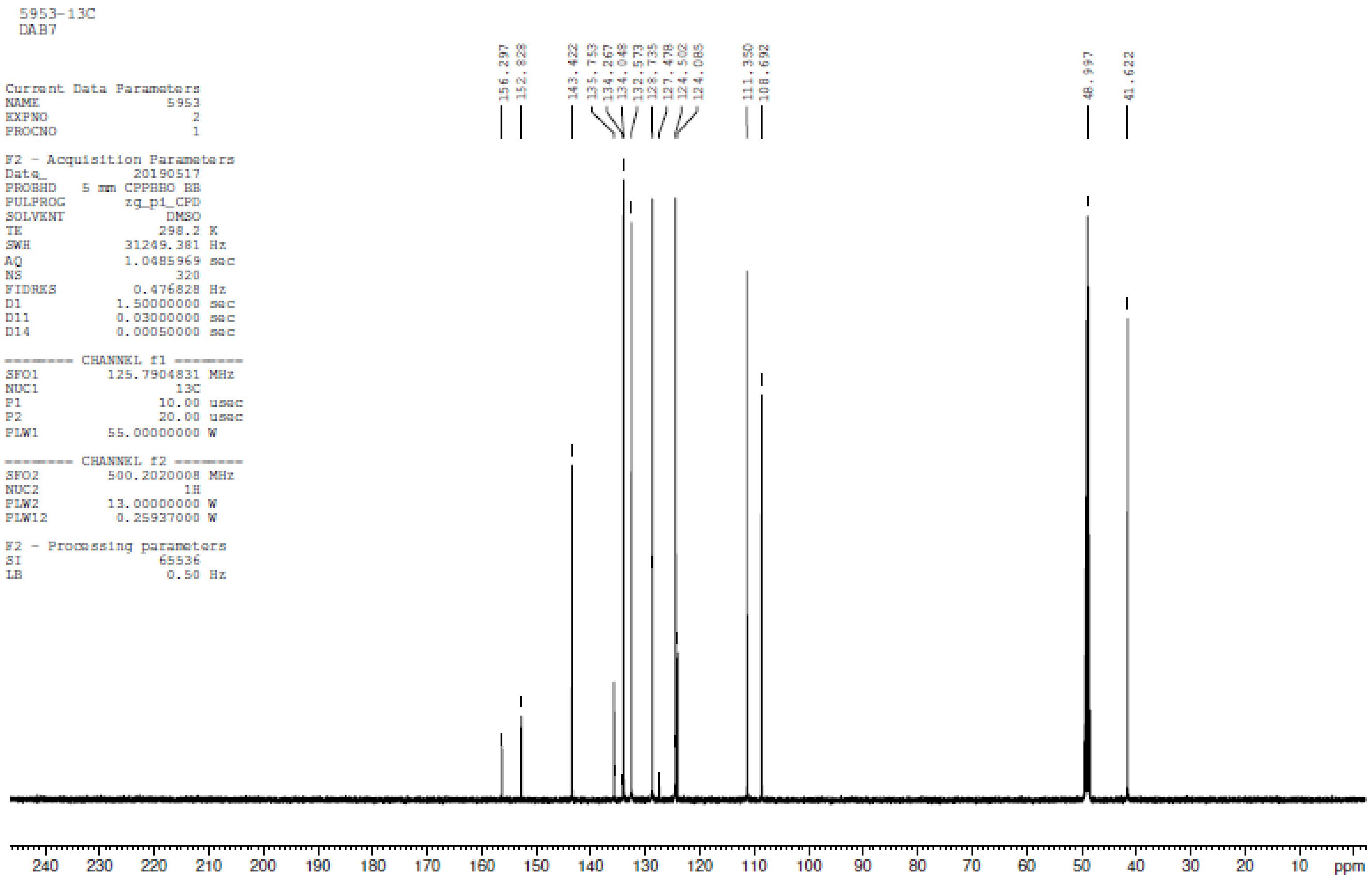
5953-13C
DAB7
Current Data Parameters
NAME 5953
EXPNO 2
PROCNO 1
F2 - Acquisition Parameters
Date_ 20190517
PROBHD 5 mm CPPBBO BB
PULPROG zg_pi_CPD
SOLVENT DMSO
TE 298.2 K
SWH 31249.381 Hz
AQ 1.0485969 sec
NS 320
FIDRES 0.476828 Hz
D1 1.50000000 sec
D11 0.03000000 sec
D14 0.00050000 sec
======== CHANNEL f1 ========
SFO1 125.7904831 MHz
NUC1 13C
P1 10.00 usec
P2 20.00 usec
PLW1 55.00000000 W
======== CHANNEL f2 ========
SFO2 500.2020008 MHz
NUC2 1H
PLW2 13.00000000 W
PLW12 0.25937000 W
F2 - Processing parameters
SI 65536
LB 0.50 Hz
156.297
152.828
143.422
135.753
134.267
134.048
132.573
128.735
127.478
124.502
124.085
111.350
108.692
48.997
41.622
240 230 220 210 200 190 180 170 160 150 140 130 120 110 100 90 80 70 60 50 40 30 20 10 ppm

Compound 8

5954-1H
DAB8

Current Data Parameters
NAME 5954
EXPNO 1
PROCNO 1

F2 - Acquisition Parameters
Date_ 20190517
PROBHD 5 mm CPPBBO BB
PULPROG zg30
SOLVENT DMSO
TE 298.2 K
SWH 10000.000 Hz
AQ 3.2767999 sec
NS 8
FIDRES 0.152588 Hz
D1 0 sec

======== CHANNEL f1 ========
SFO1 500.2030889 MHz
NUC1 1H
P1 11.30 usec
PLW1 13.69999981 W

F2 - Processing parameters
SI 65536
LB 0 Hz

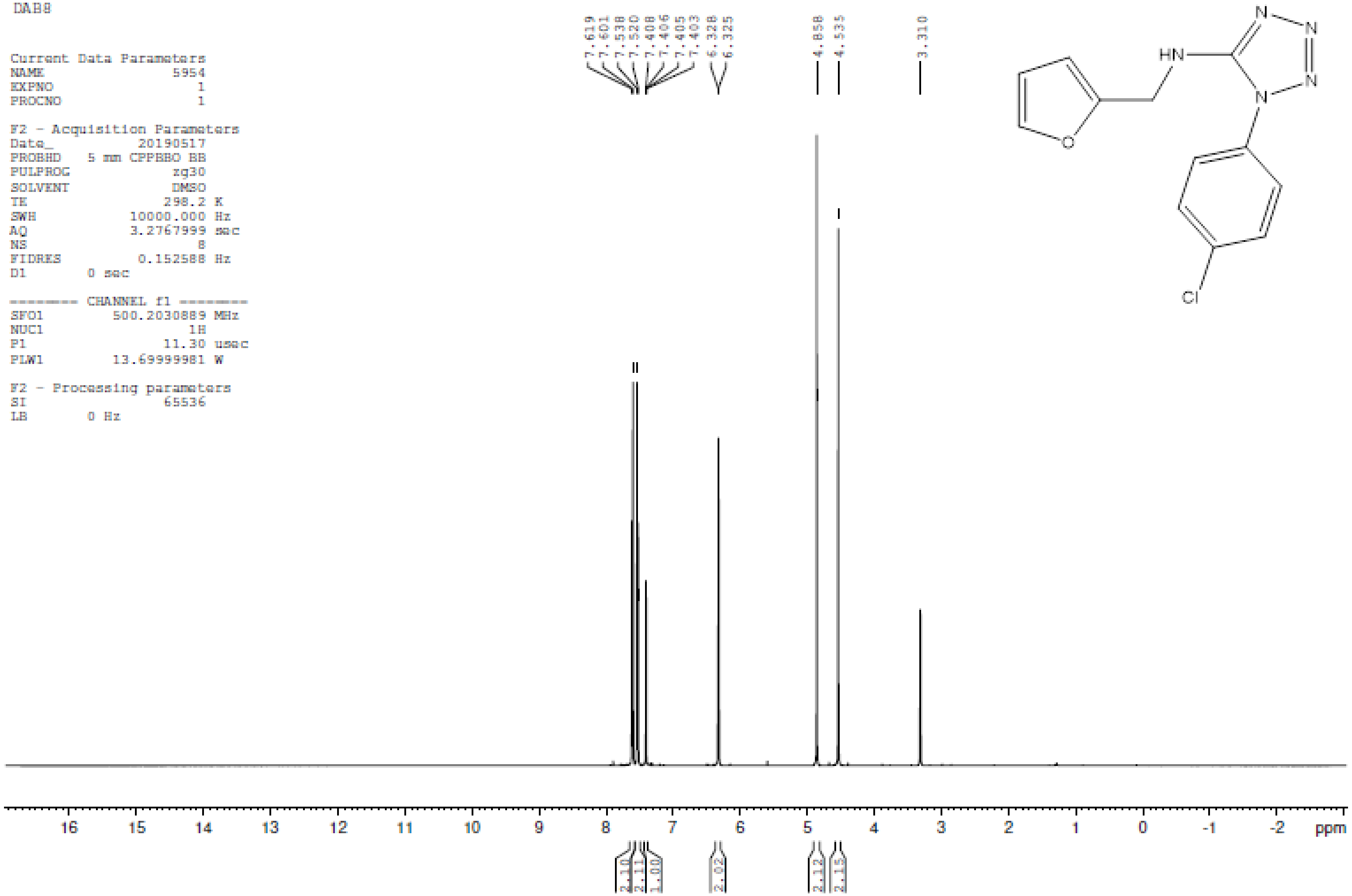

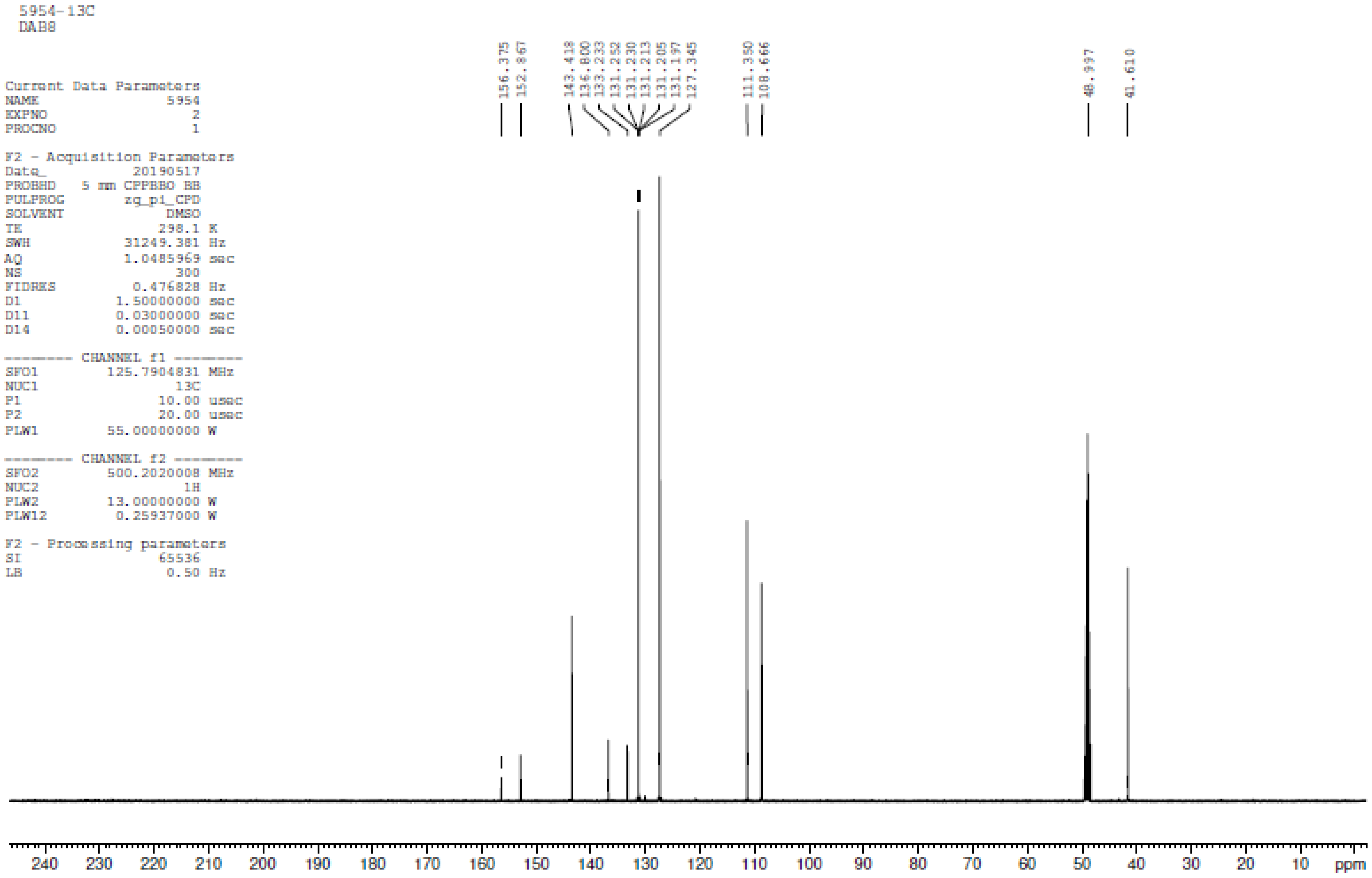
5954-13C
DAB8
Current Data Parameters
NAME 5954
EXPNO 2
PROCNO 1
F2 - Acquisition Parameters
Date_ 20190517
PROBHD 5 mm CPPBBO BB
PULPROG zg_pi_CPD
SOLVENT DMSO
TE 298.1 K
SWH 31249.381 Hz
AQ 1.0485969 sec
NS 300
FIDRES 0.476828 Hz
D1 1.50000000 sec
D11 0.03000000 sec
D14 0.00050000 sec
======== CHANNEL f1 ========
SFO1 125.7904831 MHz
NUC1 13C
P1 10.00 usec
P2 20.00 usec
PLW1 55.00000000 W
======== CHANNEL f2 ========
SFO2 500.2020008 MHz
NUC2 1H
PLW2 13.00000000 W
PLW12 0.25937000 W
F2 - Processing parameters
SI 65536
LB 0.50 Hz
156.375
152.867
143.418
136.800
133.233
131.252
131.230
131.213
131.205
131.197
127.345
111.350
108.666
48.997
41.610
240 230 220 210 200 190 180 170 160 150 140 130 120 110 100 90 80 70 60 50 40 30 20 10 ppm

Compound 9

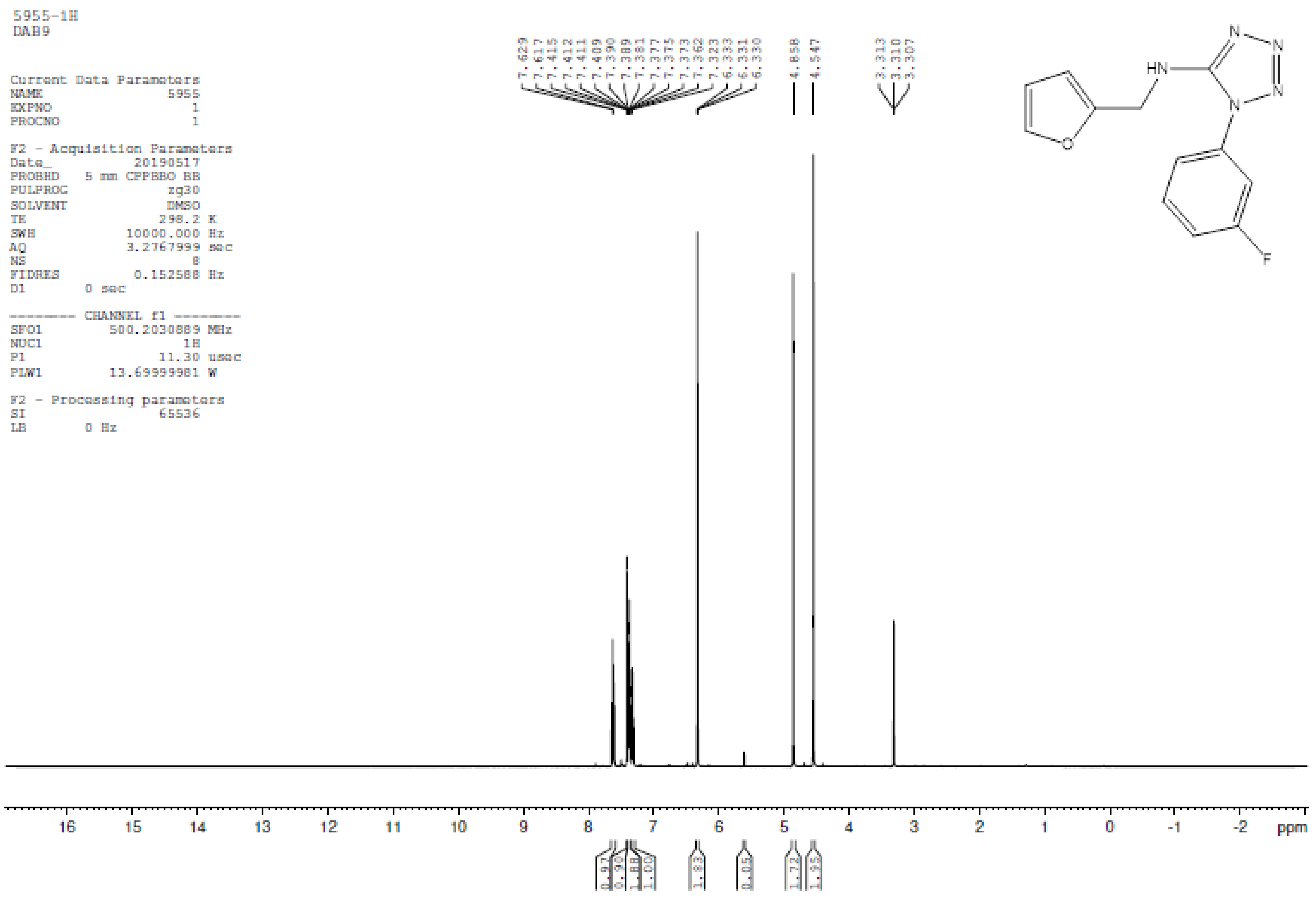
5955-1H
DAB9
Current Data Parameters
NAME 5955
EXPNO 1
PROCNO 1
F2 - Acquisition Parameters
Date_ 20190517
PROBHD 5 mm CPPBBO BB
PULPROG zg30
SOLVENT DMSO
TE 298.2 K
SWH 10000.000 Hz
AQ 3.2767999 sec
NS 8
FIDRES 0.152588 Hz
D1 0 sec
======== CHANNEL f1 ========
SFO1 500.2030889 MHz
NUC1 1H
P1 11.30 usec
PLW1 13.69999981 W
F2 - Processing parameters
SI 65536
LB 0 Hz
7.629
7.617
7.415
7.412
7.411
7.409
7.390
7.389
7.381
7.377
7.375
7.373
7.362
7.323
6.333
6.331
6.330
4.858
4.547
3.313
3.310
3.307
HN
N
N
N
N
O
F
16
15
14
13
12
11
10
9
8
7
6
5
4
3
2
1
0
-1
-2
ppm
0.97
0.90
1.88
1.00
1.83
0.05
1.72
1.95

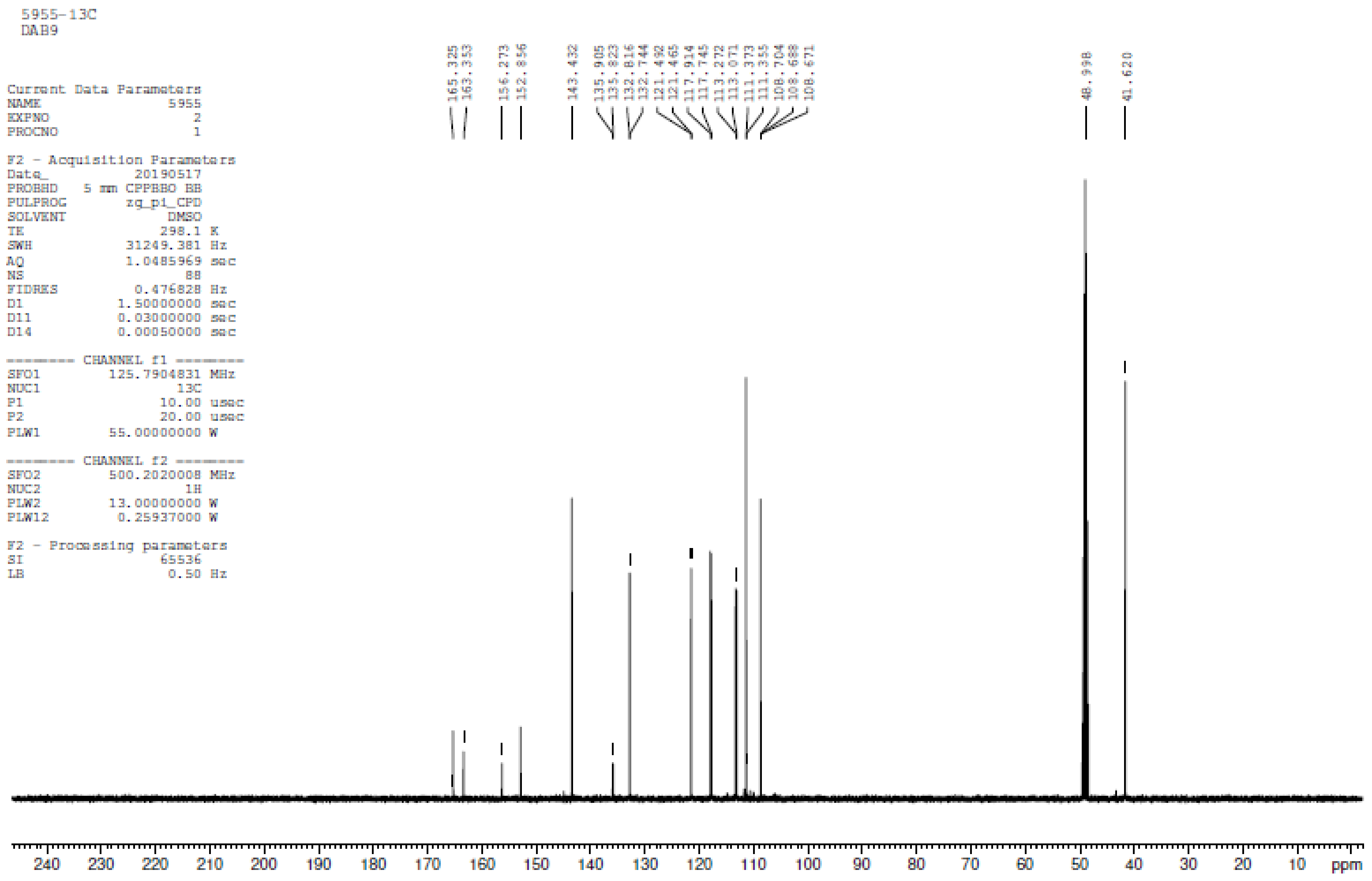
5955-13C
DAB9
Current Data Parameters
NAME 5955
EXPNO 2
PROCNO 1
F2 - Acquisition Parameters
Date_ 20190517
PROBHD 5 mm CPPBBO BB
PULPROG zg_pi_CPD
SOLVENT DMSO
TE 298.1 K
SWH 31249.381 Hz
AQ 1.0485969 sec
NS 88
FIDRES 0.476828 Hz
D1 1.50000000 sec
D11 0.03000000 sec
D14 0.00050000 sec
======== CHANNEL f1 ========
SFO1 125.7904831 MHz
NUC1 13C
P1 10.00 usec
P2 20.00 usec
PLW1 55.00000000 W
======== CHANNEL f2 ========
SFO2 500.2020008 MHz
NUC2 1H
PLW2 13.00000000 W
PLW12 0.25937000 W
F2 - Processing parameters
SI 65536
LB 0.50 Hz
165.325
163.353
156.273
152.856
143.432
135.905
135.823
132.816
132.744
121.492
121.465
117.914
117.745
113.272
113.071
111.373
111.355
108.704
108.688
108.671
48.998
41.620
240 230 220 210 200 190 180 170 160 150 140 130 120 110 100 90 80 70 60 50 40 30 20 10 ppm

Compound 10

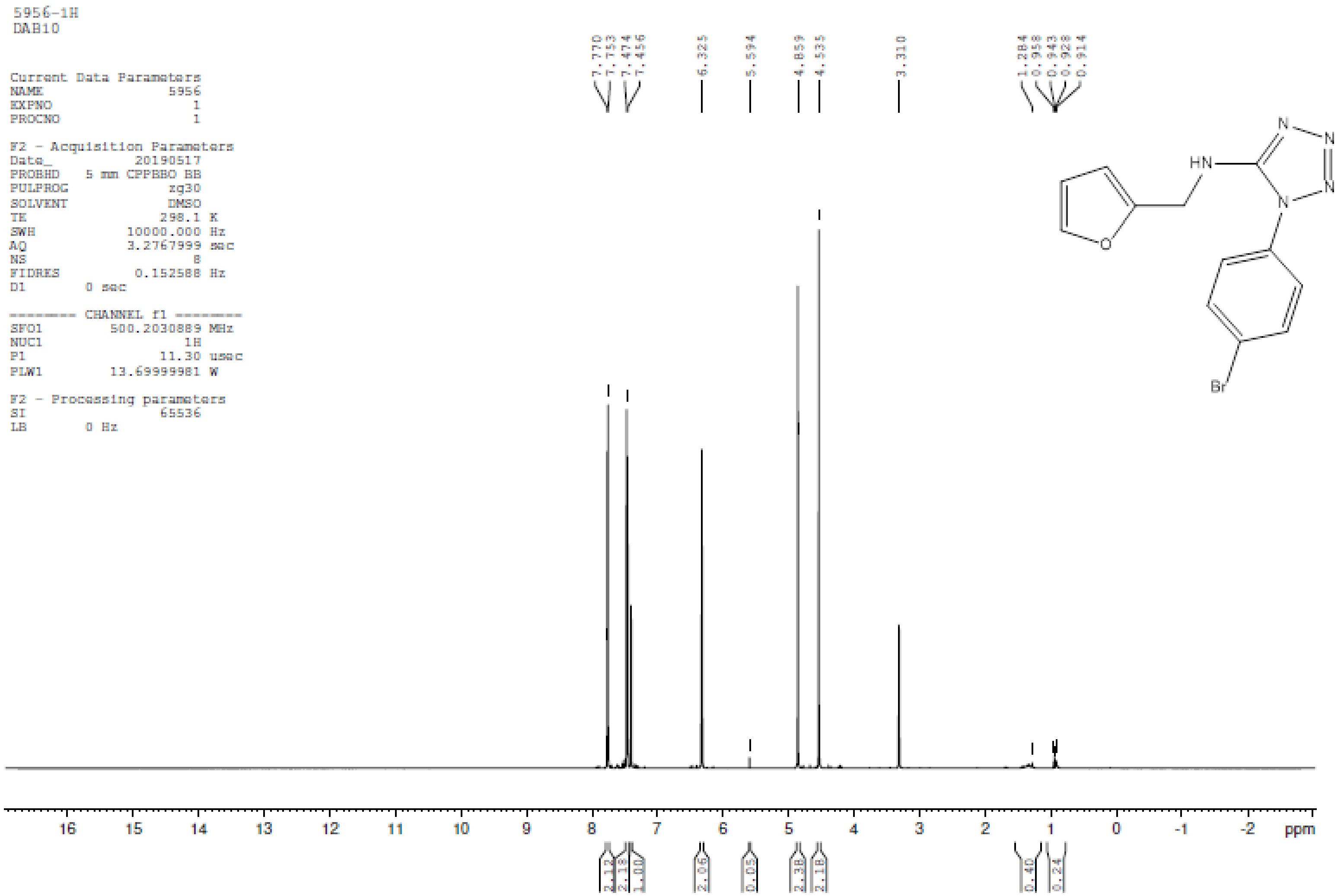
5956-1H
DAB10
Current Data Parameters
NAME 5956
EXPNO 1
PROCNO 1
F2 - Acquisition Parameters
Date_ 20190517
PROBHD 5 mm CPPBBO BB
PULPROG zg30
SOLVENT DMSO
TE 298.1 K
SWH 10000.000 Hz
AQ 3.2767999 sec
NS 8
FIDRES 0.152588 Hz
D1 0 sec
======== CHANNEL f1 ========
SFO1 500.2030889 MHz
NUC1 1H
P1 11.30 usec
PLW1 13.69999981 W
F2 - Processing parameters
SI 65536
LB 0 Hz
7.770
7.753
7.474
7.456
6.325
5.594
4.859
4.535
3.310
1.284
0.958
0.943
0.928
0.914
HN
N
N
N
N
O
Br
16
15
14
13
12
11
10
9
8
7
6
5
4
3
2
1
0
-1
-2
ppm
2.12
2.18
1.00
2.06
0.05
2.38
2.18
0.40
0.24

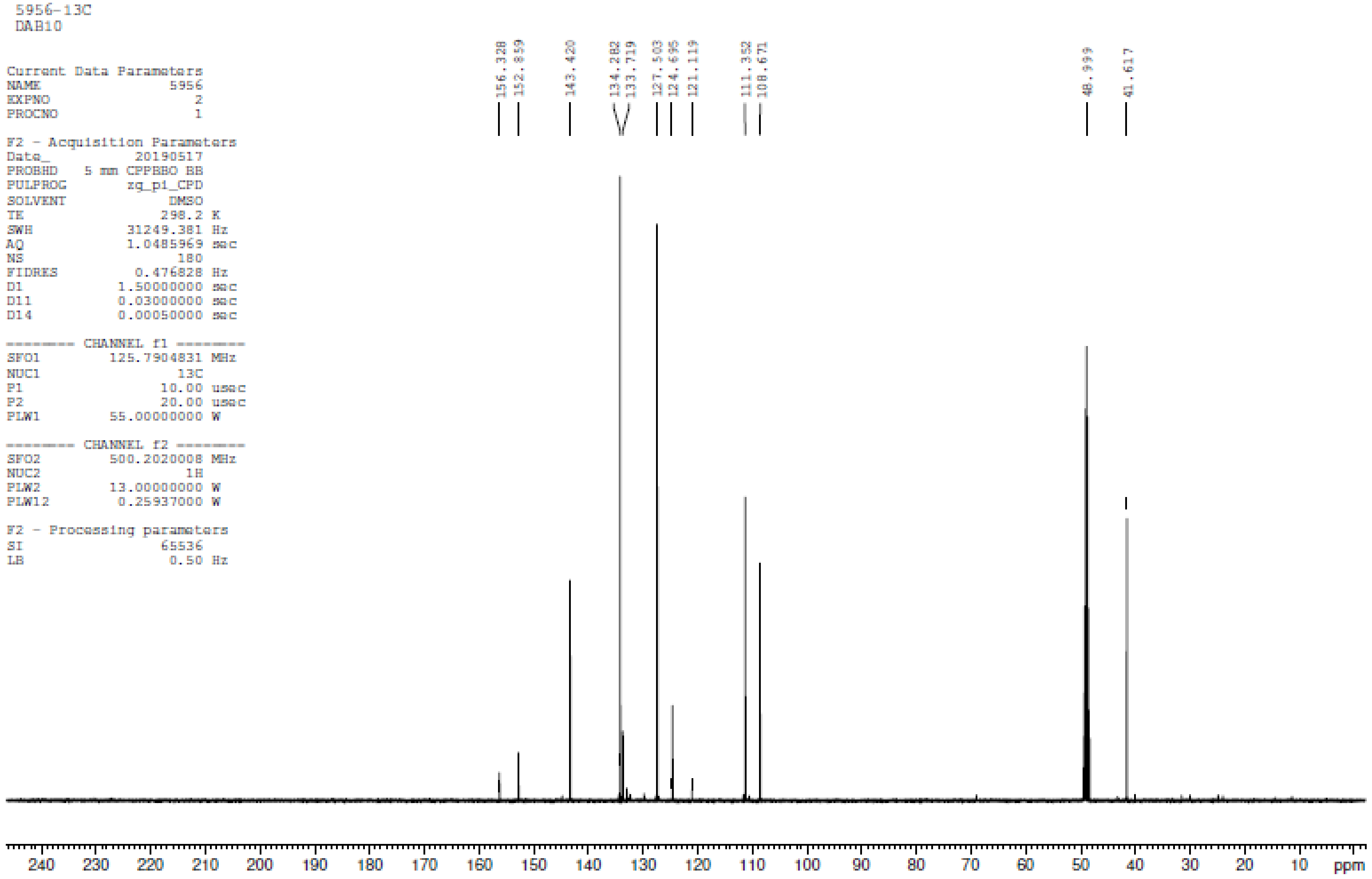
5956-13C
DAB10
Current Data Parameters
NAME 5956
EXPNO 2
PROCNO 1
F2 - Acquisition Parameters
Date_ 20190517
PROBHD 5 mm CPPBBO BB
PULPROG zg_pi_CPD
SOLVENT DMSO
TE 298.2 K
SWH 31249.381 Hz
AQ 1.0485969 sec
NS 180
FIDRES 0.476828 Hz
D1 1.50000000 sec
D11 0.03000000 sec
D14 0.00050000 sec
======== CHANNEL f1 ========
SFO1 125.7904831 MHz
NUC1 13C
P1 10.00 usec
P2 20.00 usec
PLW1 55.00000000 W
======== CHANNEL f2 ========
SFO2 500.2020008 MHz
NUC2 1H
PLW2 13.00000000 W
PLW12 0.25937000 W
F2 - Processing parameters
SI 65536
LB 0.50 Hz
156.328
152.859
143.420
134.282
133.719
127.503
124.695
121.119
111.352
108.671
48.999
41.617
240 230 220 210 200 190 180 170 160 150 140 130 120 110 100 90 80 70 60 50 40 30 20 10 ppm

Compound 11

5957-1H
DAB11

Current Data Parameters
NAME 5957
EXPNO 1
PROCNO 1

F2 - Acquisition Parameters
Date_ 20190517
PROBHD 5 mm CPPBBO BB
PULPROG zg30
SOLVENT DMSO
TE 298.1 K
SWH 10000.000 Hz
AQ 3.2767999 sec
NS 8
FIDRES 0.152588 Hz
D1 0 sec

======== CHANNEL f1 ========
SFO1 500.2030889 MHz
NUC1 1H
P1 11.30 usec
PLW1 13.69999981 W

F2 - Processing parameters
SI 65536
LB 0 Hz

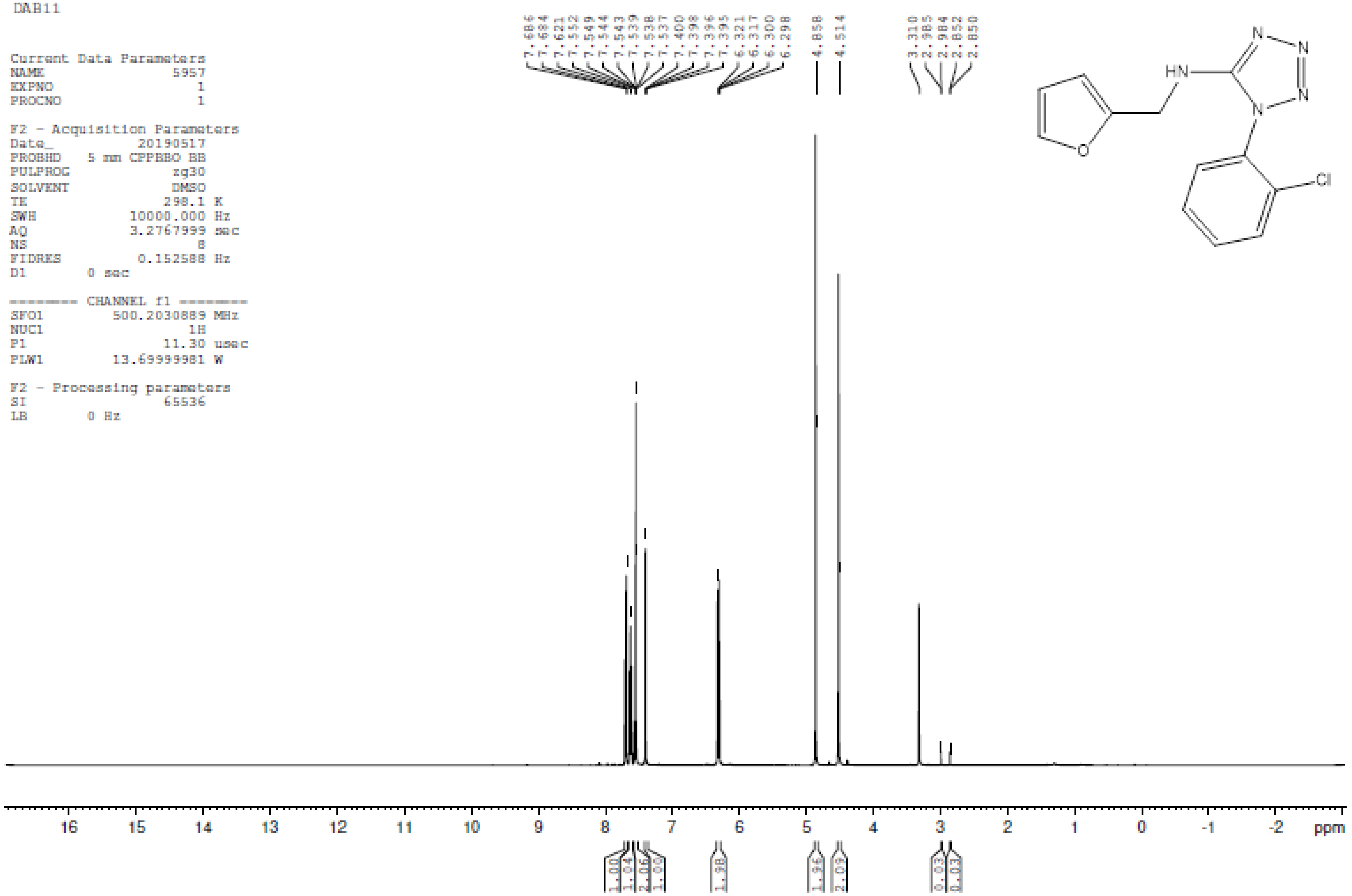

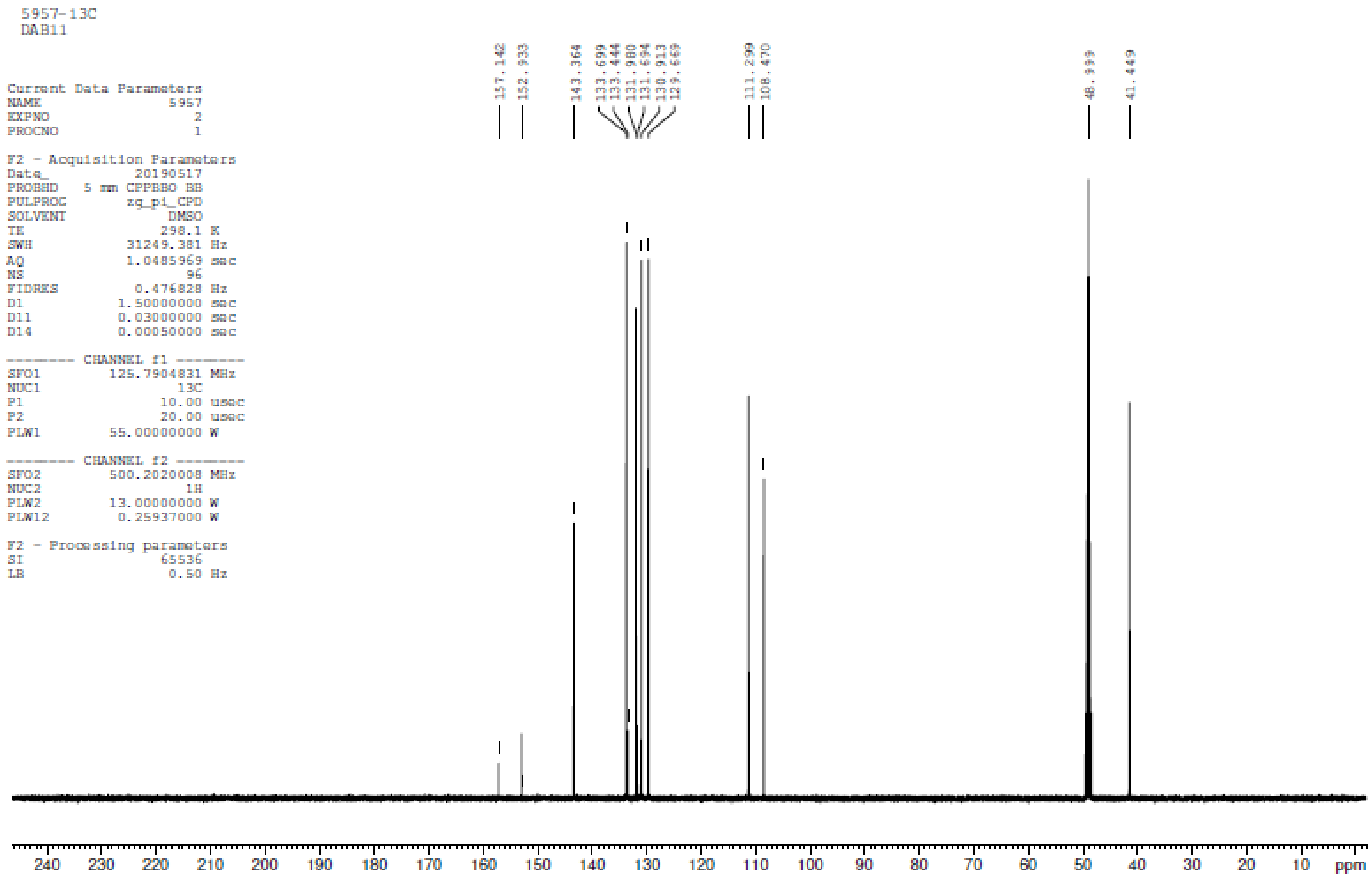
5957-13C
DAB11
Current Data Parameters
NAME 5957
EXPNO 2
PROCNO 1
F2 - Acquisition Parameters
Date_ 20190517
PROBHD 5 mm CPPBBO BB
PULPROG zg_pi_CPD
SOLVENT DMSO
TE 298.1 K
SWH 31249.381 Hz
AQ 1.0485969 sec
NS 96
FIDRES 0.476828 Hz
D1 1.50000000 sec
D11 0.03000000 sec
D14 0.00050000 sec
======== CHANNEL f1 ========
SFO1 125.7904831 MHz
NUC1 13C
P1 10.00 usec
P2 20.00 usec
PLW1 55.00000000 W
======== CHANNEL f2 ========
SFO2 500.2020008 MHz
NUC2 1H
PLW2 13.00000000 W
PLW12 0.25937000 W
F2 - Processing parameters
SI 65536
LB 0.50 Hz
157.142
152.933
143.364
133.699
133.444
131.980
131.694
130.913
129.669
111.299
108.470
48.999
41.449
240 230 220 210 200 190 180 170 160 150 140 130 120 110 100 90 80 70 60 50 40 30 20 10 ppm

Compound 12

5958-1H
DAB12

Current Data Parameters
NAME 5958
EXPNO 1
PROCNO 1

F2 - Acquisition Parameters
Date_ 20190517
PROBHD 5 mm CPPBBO BB
PULPROG zg30
SOLVENT DMSO
TE 298.2 K
SWH 10000.000 Hz
AQ 3.2767999 sec
NS 8
FIDRES 0.152588 Hz
D1 0 sec

======== CHANNEL f1 ========
SFO1 500.2030889 MHz
NUC1 1H
P1 11.30 usec
PLW1 13.69999981 W

F2 - Processing parameters
SI 65536
LB 0 Hz

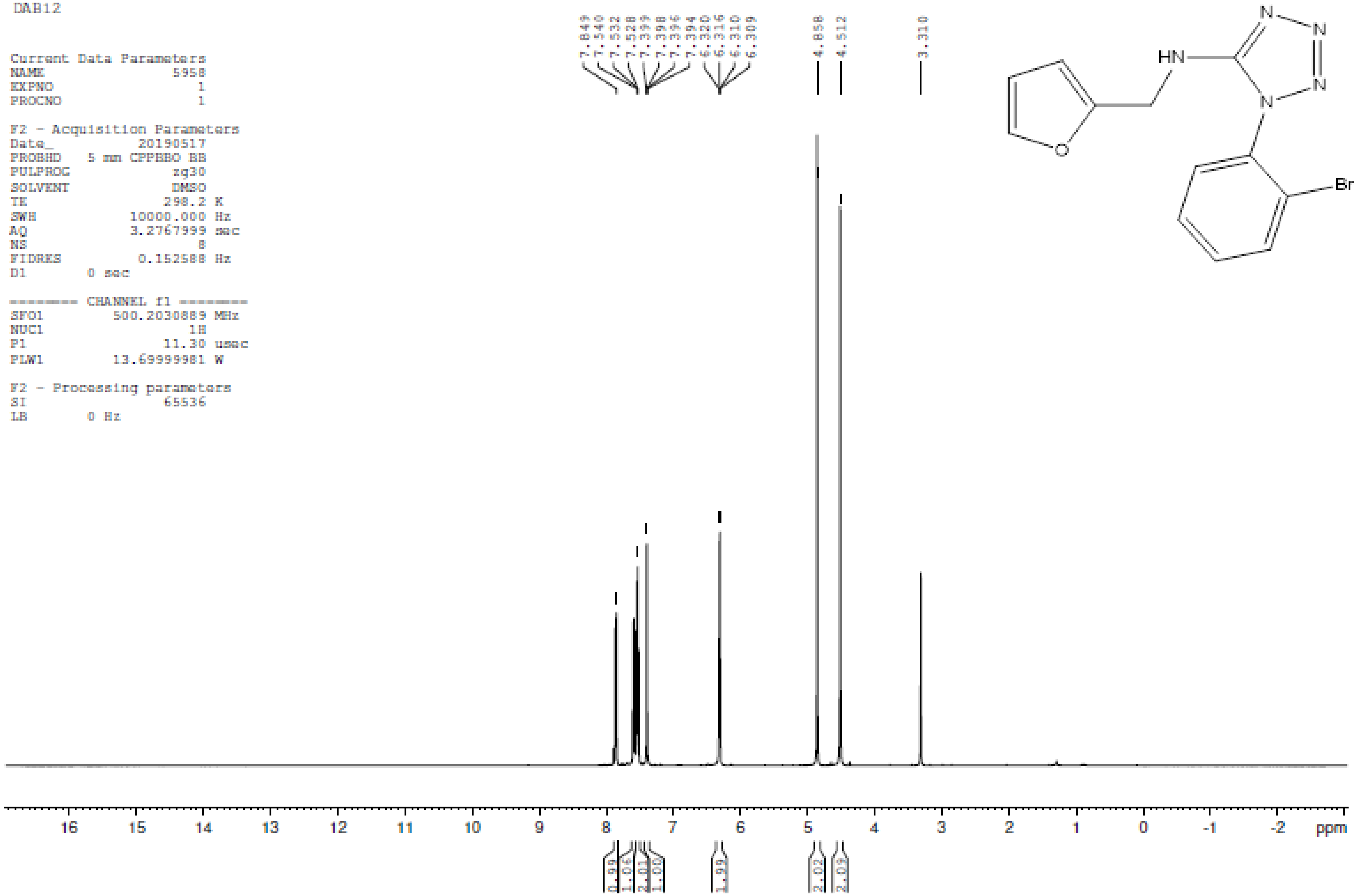

5958-13C
DAB12

Current Data Parameters
NAME 5958
EXPNO 2
PROCNO 1

F2 - Acquisition Parameters
Date_ 20190517
PROBHD 5 mm CPPBBO BB
PULPROG zg_pi_CPD
SOLVENT DMSO
TE 298.2 K
SWH 31249.381 Hz
AQ 1.0485969 sec
NS 292
FIDRES 0.476828 Hz
D1 1.50000000 sec
D11 0.03000000 sec
D14 0.00050000 sec

======== CHANNEL f1 ========
SFO1 125.7904831 MHz
NUC1 13C
P1 10.00 usec
P2 20.00 usec
PLW1 55.00000000 W

======== CHANNEL f2 ========
SFO2 500.2020008 MHz
NUC2 1H
PLW2 13.00000000 W
PLW12 0.25937000 W

F2 - Processing parameters
SI 65536
LB 0.50 Hz

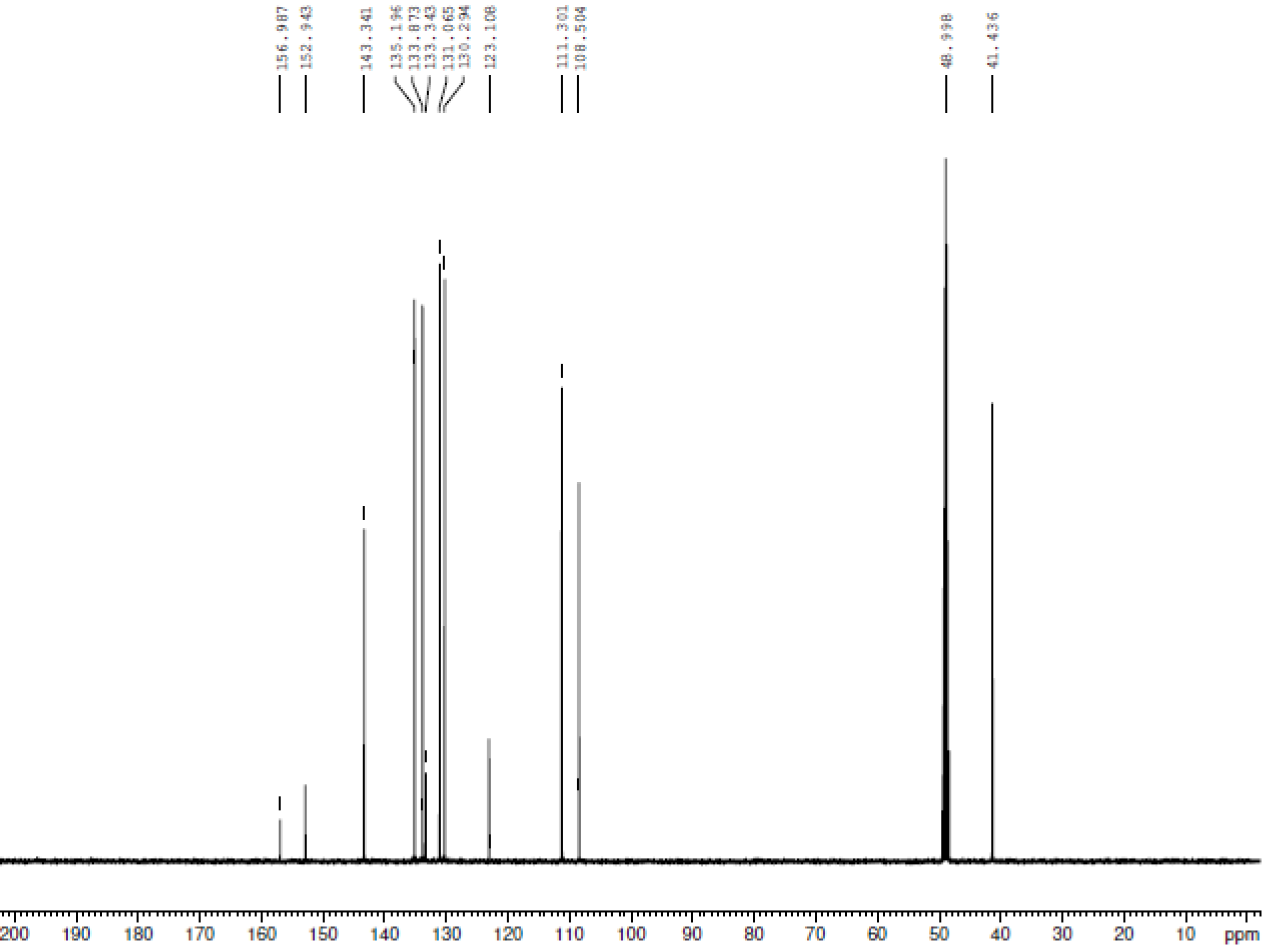

Supplement: Supplementary file 1 [file molecules-26-00323-s001.zip › supplementary_files/NMR file.pdf]
