# Supplementary material for: Synthetic Transition from Thiourea-Based Compounds to Tetrazole Derivatives: Structure and Biological Evaluation of Synthesized New N-(Furan-2-ylmethyl)-1H-tetrazol-5-amine Derivatives
Source: Molecules. 2021 Jan 10;26(2):323. doi: 10.3390/molecules26020323 (PMC7827014; doi:10.3390/molecules26020323)
Supplement: Supplementary file 1 [file molecules-26-00323-s001.zip › supplementary_files/Table S2.pdf]

Supplementary material

Synthetic transition from thiourea-based compounds to tetrazole derivatives. Structure and biological evaluation of synthesized new *N*-(furan-2-ylmethyl)-1*H*-tetrazol-5-amine derivatives.

Daniel Szulczyk[a*], Anna Bielenica[a], Piotr Roszkowski[c], Michał A. Dobrowolski[c], Wioletta Olejarz[b], Sebastian Kmiecik[d], Małgorzata Podsiad[a] and Marta Struga[a].

[a]*Chair and Department of Biochemistry, Medical University of Warsaw, 02-097 Warszawa, Poland*

[b]*Department of Biochemistry and Pharmacogenomics, Faculty of Pharmacy, Medical University of Warsaw, 02-097 Warszawa, Poland*

[c]*Faculty of Chemistry, University of Warsaw, Pasteura 1, 02-093 Warszawa, Poland*

[d]*Biological and Chemical Research Centre, Faculty of Chemistry, University of Warsaw, 02-089 Warsaw, Poland*

In vitro evaluation of antitubercular properties against *M. tuberculosis* H37Rv strain and two “wild-type” mycobacteria isolated from tuberculosis patients: multidrug-resistant Spec. 210 [with resistance to p-aminosalicylic acid (PAS), isoniazid (INH), ethambutol (EMB) and rifampicin (RMP)] and Spec. 192, fully susceptible to established tuberculostatics.

**Compound**	**Minimal inhibitory concentration (µg/ml)**		
	M. tuberculosis **$H_{37}R_v$**	***M. tuberculosis INH resistant (Spec. 210)***	***M. tuberculosis INH susceptible (Spec. 192)***
**1.**	256	256	256
**2.**	>512	512	>512
**3.**	>512	512	>512
**4.**	>512	256	512
**5.**	>512	>512	>512
**6.**	512	256	512
**7.**	512	256	512
**8.**	256	256	512
**9.**	256	256	256
**10.**	>512	512	>512
**11.**	512	256	256
**12.**	512	256	512
**INH**	≤0,0625	2	≤0,0625
