# Supplementary material for: Synthetic Transition from Thiourea-Based Compounds to Tetrazole Derivatives: Structure and Biological Evaluation of Synthesized New N-(Furan-2-ylmethyl)-1H-tetrazol-5-amine Derivatives
Source: Molecules. 2021 Jan 10;26(2):323. doi: 10.3390/molecules26020323 (PMC7827014; doi:10.3390/molecules26020323)
Supplement: Supplementary file 1 [file molecules-26-00323-s001.zip › supplementary_files/Table S3.pdf]

Supplementary material

Synthetic transition from thiourea-based compounds to tetrazole derivatives. Structure and biological evaluation of synthesized new *N*-(furan-2-ylmethyl)-1*H*-tetrazol-5-amine derivatives.

Daniel Szulczyk[a*], Anna Bielenica[a], Piotr Roszkowski[c], Michał A. Dobrowolski[c], Wioletta Olejarz[b], Sebastian Kmiecik[d], Małgorzata Podsiad[a] and Marta Struga[a].

[a]*Chair and Department of Biochemistry, Medical University of Warsaw, 02-097 Warszawa, Poland*

[b]*Department of Biochemistry and Pharmacogenomics, Faculty of Pharmacy, Medical University of Warsaw, 02-097 Warszawa, Poland*

[c]*Faculty of Chemistry, University of Warsaw, Pasteura 1, 02-093 Warszawa, Poland*

[d]*Biological and Chemical Research Centre, Faculty of Chemistry, University of Warsaw, 02-089 Warsaw, Poland*

MTT – 72 h

Antiproliferative activity of tested compounds. Data are given as IC_{50} [μM] and SI.

Compound	Cancer cells						Normal cells
	**Caco-2**		**A549**		**HTB-140**		**HaCaT**
	IC_{50}	SI	IC_{50}	SI	IC_{50}	SI	IC_{50}
**1**	> 100	-	> 100	-	> 100	-	> 100
**2**	> 100	-	> 100	-	> 100	-	> 100
**3**	> 100	-	> 100	-	> 100	-	> 100
**4**	> 100	-	> 100	-	> 100	-	> 100
**5**	> 100	-	> 100	-	> 100	-	> 100
**6**	> 100	-	> 100	-	> 100	-	> 100
**7**	> 100	-	> 100	-	> 100	-	> 100
**8**	> 100	-	> 100	-	> 100	-	> 100
**9**	> 100	-	> 100	-	> 100	-	> 100
**10**	> 100	-	> 100	-	> 100	-	> 100
**11**	> 100	-	> 100	-	> 100	-	> 100
**12**	> 100	-	> 100	-	> 100	-	> 100
cisplatin	1,68 ± 0,94	1,69	1,95 ± 0,83	1,46	1,13 ± 0,19	2,51	2,84 ± 1,06
doxorubicin	0,53 ± 0,23	2,06	0,63 ± 0,21	1,73	0,47 ±0 ,18	2,32	1,09 ± 0,23

The IC_{50} value is defined as the concentration of a compound that corresponds to a 50% growth inhibition. Human immortal keratinocyte cell line from adult human skin (HaCaT), Human colorectal adenocarcinoma (CaCo-2), Human epithelial lung carcinoma cell line (A549) and Human melanoma cell line (HTB-140). Data are expressed as mean ± SD. The SI (Selectivity Index) was calculated for each compound using formula: SI = IC_{50} for normal cel line / IC_{50} cancer cel line.
