# Supplementary material for: Synthetic Transition from Thiourea-Based Compounds to Tetrazole Derivatives: Structure and Biological Evaluation of Synthesized New N-(Furan-2-ylmethyl)-1H-tetrazol-5-amine Derivatives
Source: Molecules. 2021 Jan 10;26(2):323. doi: 10.3390/molecules26020323 (PMC7827014; doi:10.3390/molecules26020323)
Supplement: Supplementary file 1 [file molecules-26-00323-s001.zip › supplementary_files/X-ray studies file.pdf]

Supplementary material

Synthetic transition from thiourea-based compounds to tetrazole derivatives. Structure and biological evaluation of synthesized new *N*-(furan-2-ylmethyl)-1*H*-tetrazol-5-amine derivatives.

Daniel Szulczyk[a*], Anna Bielenica[a], Piotr Roszkowski[c], Michał A. Dobrowolski[c], Wioletta Olejarz[b], Sebastian Kmiecik[d], Małgorzata Podsiad[a] and Marta Struga[a].

[a]*Chair and Department of Biochemistry, Medical University of Warsaw, 02-097 Warszawa, Poland*

[b]*Department of Biochemistry and Pharmacogenomics, Faculty of Pharmacy, Medical University of Warsaw, 02-097 Warszawa, Poland*

[c]*Faculty of Chemistry, University of Warsaw, Pasteura 1, 02-093 Warszawa, Poland*

[d]*Biological and Chemical Research Centre, Faculty of Chemistry, University of Warsaw, 02-089 Warsaw, Poland*

Table S1. Crystal data and structure refinement for **8a** and **8**.

Compound	**8a**	**8**
Empirical formula	$C_{12}H_{11}Cl_1N_2O_1S_1$	$C_{12}H_{10}Cl_1N_5O_1$
Formula weight	266.74	275.70
Temperature [K]	100	100
Space group	$P2_1/c$	$P2_1$
Unit cell dimensions		
a [Å]	10.1309(3)	7.2890(5)
b [Å]	12.3811(4)	6.2742(4)
c [Å]	9.7048(3)	13.2757(8)
α [°]	90	90
β [°]	96.345(2)	90.073(6)
γ [°]	90	90
Volume *V* [Å^3]	1209.83(7)	607.13(7)
Z [molecules/cell]	4	2
$D_{calculated}$ [g cm^{-3}]	1.464	1.508
Absorption coefficient μ/ mm^{-1}	0.472	0.314

θ range for data collection [°]	2.61-25.00	2.794-26.369
Limiting indices	-12 < = h = > 12	-9 < = h = > 9
	-14 < = k = > 14	-7 < = k = > 7
	-11 < = l = > 11	-16 < = l = > 16
Reflections collected/unique	63087/2127	7932/2469
Data/parameters	2127/162	2469/175
Goodness of Fit	1.163	1.033
Final R index ($I > 2\sigma$)	0.0278	0.0381
wR^2	0.0571	0.0737
Flack parameter	-	0.01(4)
Largest diff. Peak and hole [$Å^{-3}$]	0.255 and -0.261	0.257 and -0.197
CCDC	2047281	2047282

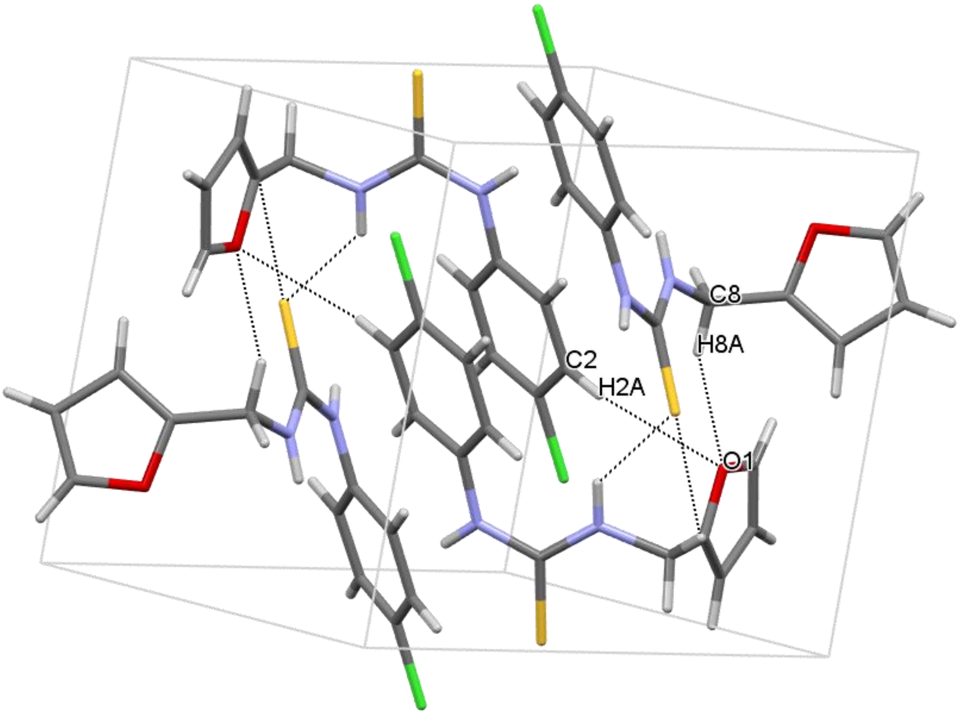

Figure S1. Intermolecular interactions in the crystal lattice of **8a**.

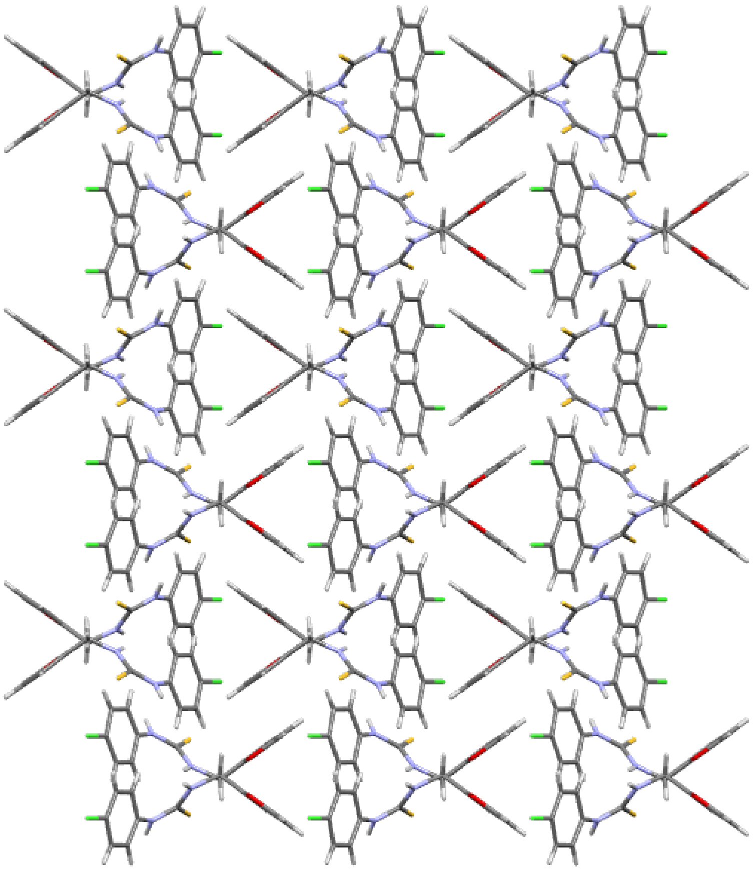

Figure S2. Fragment of crystal lattice 3x3 unit cells along [001] direction for **8a**.

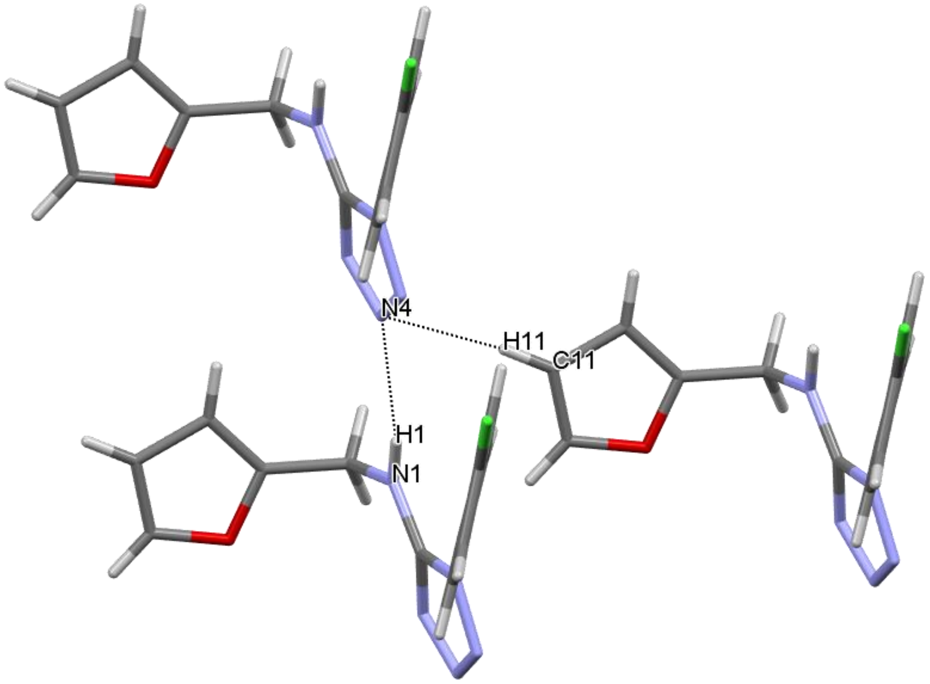

Figure S3. Intermolecular interactions in the crystal lattice of **8**.

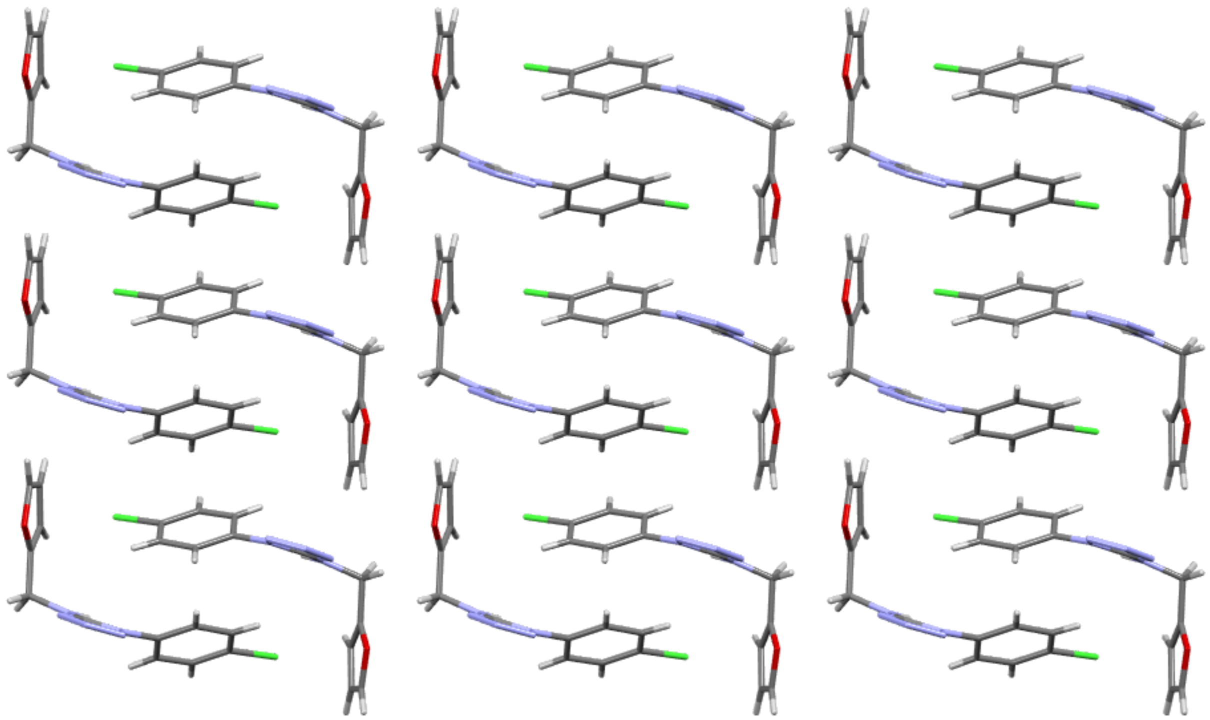

Figure S4. Fragment of crystal lattice 3x3 unit cells along [010] direction for **8**.
